# Glutamate controls vessel-associated migration of GABA interneurons from the pial migratory route via NMDA receptors and endothelial protease activation

**DOI:** 10.1007/s00018-019-03248-5

**Published:** 2019-08-07

**Authors:** Cécile Léger, Nicolas Dupré, Caroline Aligny, Magalie Bénard, Alexis Lebon, Vincent Henry, Michelle Hauchecorne, Ludovic Galas, Thierry Frebourg, Philippe Leroux, Denis Vivien, Maryline Lecointre, Stéphane Marret, Bruno J. Gonzalez

**Affiliations:** 1 Normandie University, UNIROUEN, INSERM U1245 and Rouen University Hospital, Department of Neonatal Paediatrics and Intensive Care, F 76000, Normandy Centre for Genomic and Personalized Medicine, Rouen, France; 2 Inserm, Université Caen-Normandie, Inserm, UMR-S U1237 “Physiopathology and Imaging of Neurological Disorders” (PhIND), GIP Cyceron, Caen, France; 3 Department of Clinical Research, Caen University Hospital, CHU Caen, Caen, France; 4 Normandie University, UNIROUEN, INSERM, PRIMACEN, Rouen, France

**Keywords:** Endothelial cells, GABA interneuron, Migration, MMP-9, NMDAR, t-PA

## Abstract

During cortex development, fine interactions between pyramidal cells and migrating GABA neurons are required to orchestrate correct positioning of interneurons, but cellular and molecular mechanisms are not yet clearly understood. Functional and age-specific expression of NMDA receptors by neonate endothelial cells suggests a vascular contribution to the trophic role of glutamate during cortical development. Associating functional and loss-of-function approaches, we found that glutamate stimulates activity of the endothelial proteases MMP-9 and t-PA along the pial migratory route (PMR) and radial cortical microvessels. Activation of MMP-9 was NMDAR-dependent and abrogated in t-PA^−/−^ mice. Time-lapse recordings revealed that glutamate stimulated migration of GABA interneurons along vessels through an NMDAR-dependent mechanism. In Gad67-GFP mice, t-PA invalidation and in vivo administration of an MMP inhibitor impaired positioning of GABA interneurons in superficial cortical layers, whereas Grin1 endothelial invalidation resulted in a strong reduction of the thickness of the pial migratory route, a marked decrease of the glutamate-induced MMP-9-like activity along the PMR and a depopulation of interneurons in superficial cortical layers. This study supports that glutamate controls the vessel-associated migration of GABA interneurons by regulating the activity of endothelial proteases. This effect requires endothelial NMDAR and is t-PA-dependent. These neurodevelopmental data reinforce the debate regarding safety of molecules with NMDA-antagonist properties administered to preterm and term neonates.

**Electronic supplementary material:**

The online version of this article (10.1007/s00018-019-03248-5) contains supplementary material, which is available to authorized users.

## Introduction

At the cellular and molecular levels, there is increasing evidence that, in the developing cortex, endothelial cells are not phenotypically or regionally similar [1, 2]. For example, in the mouse, the transcriptomic and proteomic expression profiles of cortical microvessels revealed age-dependent specificities that will drastically change postnatally because of strong remodeling of the extracellular matrix, cell adhesion, and junction protein expression [1]. Similarly, at a given developmental stage, endothelial cells of the pial surface have phenotypic identities distinct from those of the periventricular vascular network, suggesting distinct contributions to the developing brain [2]. Consistent with this notion, meninges have been shown to release chemoattractive factors such as the chemokine CXCl12 essential in the control of nervous cell migration [3, 4]. Interestingly, it has been shown that there are region-specific and age-specific changes in the expression of both NMDA receptor subunits and excitatory amino acid transporters by neonatal endothelial cells [5, 6] and in vitro studies showed that NMDA is able to promote secretion of two endothelial proteases, i.e., matrix metalloproteinase-9 (MMP-9) and tissue-plasminogen activator (t-PA) by cultured neonatal endothelial cells [6]. These two proteases are involved in the control of cell migration in physiological and/or pathological conditions [7, 8] and the preferential expression pattern of NMDA receptors by endothelial cells during the perinatal period coincides with the late migration of GABA interneurons [9]. Because it has been demonstrated that during cortical development, interneurons migrate tangentially while closely interacting with the vasculature of the pial migratory route (PMR) [2, 10, 11], these data suggest that the endothelial NMDA receptor would contribute in the vessel-associated interneuron migration. Consistent with this hypothesis, several recent data showed that the migration of GABAergic neurons is regulated by excitatory neurons [12] and that exposure of mouse neonate cortices to molecules with NMDA-antagonist properties such as ketamine, alcohol, or MK801 resulted in endothelial cell autophagy followed by massive apoptotic death of migrating tangential GABA interneurons present in the superficial cortical layers [13–17].

In humans, late migration of GABAergic neurons has also been reported with at least 20% of interneurons still migrating in the developing cortex at term [18]. Moreover, at the clinical level, the brains of preterm and term neonates can be frequently exposed to molecules with NMDA-antagonist properties during the early life for the purposes of neuroprotection or anesthesia [19–21]. In addition, the innocuousness of NMDA antagonists in the developing brain is not consensual and it is more and more suspected a functional link between NMDA receptor hypofunction during early life and impaired positioning of GABA interneurons that could lead to adult diseases [22–24].

In the present study, we reported that glutamate by interacting with endothelial NMDA receptors stimulates the activity of endothelial proteases, increases vessel-associated cell migration, and contributes to the positioning of GABA interneurons arising from the pial migratory route to radially enter the cortex. These data provide new evidence that the activation of the endothelial NMDA receptors by glutamate constitutes a mechanism involved in the control of the vasculo-associated migration of cortical interneurons.

## Materials and methods

### Wild-type and transgenic mice

For each mouse strain, animals were kept in a temperature-controlled room (21 ± 1 °C) with a 12-h/12-h light/dark cycle (lights on from 7:00 a.m. to 7:00 p.m.) and free access to food and water. Animal care and manipulation complied with recommendations issued by the French and European guidelines for the care and use of laboratory animals (Council Directive 86/609/EEC, license no. 21CAE035), and were supervised by an authorized investigator (B.J.G., authorization no. 7687 from the Ministère de l’Agriculture et de la Pêche). Naval Medical Research Institute mice were purchased from Janvier (Le Genest Saint Isle, France). FVB-Tg(GadGFP)45704Swn transgenic mice (#003718) were obtained from the Jackson Laboratory (Bar Harbor, ME). In this mouse strain, GABAergic interneurons arising from the GEs express enhanced green fluorescent protein under the control of the mouse Gad1 gene promoter [25]. t-PA knockout (t-PA^−/−^) mice in the C57Bl6/129 background (t-PA^−/−^ in a ratio of 87.5:12.5%) were developed and provided by the Center for Molecular and Vascular Biology at the University of Leuven in Belgium [26]. Double transgenic Gad67GFP/t-PA^−/−^ mice were developed by Inserm U1245. Floxed Grin1 mice (B6.129S4-Grin1tm2Stl/J; # 005246) were from The Jackson Laboratory. Floxed Grin1 mice allow deletion of the GluN1 subunit of the *N*-methyl-d-aspartate receptor in Cre recombinase expressing cells/tissues. VE-Cadherin-Cre (B6.FVB-Tg(Cdh5-cre)7Mlia/J; # 006137) were from The Jackson Laboratory. Cre expression can be seen in the embryo and in adult endothelium of developing and quiescent vessels.

### Chemicals

Glutamate, Coomassie blue, Hoechst 33258, isolectin-B4-TRITC/FITC, phosphate-buffered saline (PBS), bovine serum albumin (BSA), plasminogen activator inhibitor 1 (PAI-1), and Triton X-100 were from Sigma-Aldrich (Saint Quentin Fallavier, France). MK801 was obtained from Tocris (R&D, Lille, France). GM6001 was provided by Millipore SAS (Molsheim, France). SDS-PAGE Tris–glycine gel (containing 0.1% gelatin as a gelatinase substrate), renaturing buffer, developing buffer, DQ-gelatin-FITC, and DQ-casein-FITC were from Invitrogen (Cergy Pontoise, France). The gelatinase inhibitor SB-3CT was from Biomol (Lonza Sales Ltd, Basel, Switzerland). Paraformaldehyde (PFA) was obtained from Labonord (Templemars, France). Characteristics of the primary antibodies against alpha-smooth muscle actin (ACTA2), CD31, calretinin, Cre recombinase, collagen IV, doublecortin (DCX), GABA, GFAP, GFP, GluN1, MMP-9, somatostatin (SST), Gad67, and β-actin are summarized in Supplementary Table 1. The secondary antibodies Alexa Fluor 488 donkey anti-rabbit IgG (A-21206), Alexa Fluor 488 goat anti-rat (A-11006), and Alexa Fluor 594 donkey anti-goat IgG (A-11058) used for immunohistochemistry were from invitrogen.

### Genotyping by polymerase chain reaction

Mouse tail DNAs were extracted using NucleoSpin^®^ DNA Rapidlyse (Macherey–Nagel, Düren, Germany) and were amplified on a thermal cycler with the following program: 2 min, 94 °C; 2 s, 94 °C; 15 s, 65–0.5 °C per cycle decrease; 10 s, 68 °C; repeats step 2–4 for ten cycles; 10 s, 94 °C; 15 s 50 °C; 10 s, 72 °C, repeats step 3–5 for 28 cycles; 2 min, 72 °C. Genotyping was performed with primers provided by the Jackson Laboratory; Grin1 5′-AAACAGGGCTCAGTGGGTAA and 3′-GTGCTGGGATCCACATTCAT; Cre transgene 5′-GCGGTCTGGCAGTAAAAACTATC and 3′-GTGAAACAGCATTGCTGTCACTT. Electrophorese was conducted on 3.5% of agarose gels and visualized with ethidium bromide.

### Preparation of cortical microvessels and *Grin1*, *Grin2a*, and *Grin2b* qRT-PCR

Freshly isolated cortical microvessels were prepared as previously described [6]. Practically, cortices from P10 mouse neonates (20 animals per group) were homogenized in a Dounce tissue homogenizer in 10 mL molecular cellular and developmental biology (MCDB) medium 131 supplemented with 2% of FCS (fetal calf serum), 100 U/mL penicillin, and 100 mg/mL streptomycin (all from GIBCO, Invitrogen, Cergy Pontoise, France). The homogenate was centrifuged at 200*g* for 15 min at 4 °C. The pellet was suspended in 18% (w/v) dextran solution (Sigma-Aldrich, Saint Quentin Fallavier, France) and centrifuged at 2,000*g* for 45 min at 4 °C. Thereafter, it was suspended in PBS 1X and filtered using a 70 µm cell strainer (Falcon, BD Biosciences, Pont de Claix, France). The resulting microvessel filtrate was centrifuged at 200*g* for 7 min at 4 °C. The pellet was suspended in 350 μL of lysis buffer (Qiagen, Courtaboeuf, France) containing 1% β-mercaptoethanol (Sigma-Aldrich) and ceramic beads for mechanical dissociation. Total RNAs were extracted using an RNeasy Micro kit (Qiagen) in accordance with the manufacturer’s instructions. After DNase digestion, cDNA was prepared from 100 ng total RNA using the QuantiTect reverse transcription kit (Qiagen). Generation of polymerase chain reaction (PCR) products was measured in real time by incorporation of the fluorescent dye SYBR Green I using a Light Cycler 96 real-time PCR system (Roche, Mannheim, Germany). For each reaction, a master mix was prepared as follows: 1X SYBR Green buffer (Biorad), and 0.5 mM sense and antisense primers (Invitrogen). PCR products for Grin1, Grin2A and Grin2B subunits of the NMDA receptor were generated using primers described in Supplementary Table 2. All PCRs were performed using the cycle conditions: 95 °C (10 s), 60 °C (30 s), and 65–95 °C (5 s), and were run for a total of 40 cycles. PCR amplification efficiency was assessed for each primer set from the slope of a standard curve generated with serial dilutions of cortical cDNA which was close to 3.3 for all primers, indicating maximal PCR amplification efficiency. The PCR product purity was verified using dissociation curves. The cDNA amount in each sample was calculated using the comparative threshold cycle (Ct) method and expressed as 2^−ΔΔCt^ using glyceraldehyde-3-phosphate dehydrogenase (GAPDH) as an internal control.

### Preparation and treatment of cerebral slices from neonate mice

Cortex brain slices were obtained from mice on postnatal day 2 (P2). Previous studies demonstrated that this stage presented developmental and lesional characteristics similar to those described for human neonates [27, 28]. Practically, mice were euthanized by decapitation and brains were rapidly dissected to isolate the hemispheres. The neocortex was immediately placed in ice-cold artificial cerebrospinal fluid (aCSF) containing (in mM) the following: NaCL, 125; KCL, 3; CaCl_2_, 2; NaH_2_PO_4_, 1.2; NaHCO_3_, 26; and d-glucose, 10 (pH 7.4). Transverse slices (250 µm) were cut at 4 °C using a vibratome (VT1000S; Leica, Reuil-Malmaison, France), transferred into 24-well Costar plates (Cambridge, MA) containing aCSF, and incubated during a recovery period of 30 min at 37 °C in a humidified incubator with a controlled atmosphere of 5% CO_2_/95% air. Then, slices were washed with fresh aCSF and treated for 3 h at 37 °C with glutamate (25, 50, 100 µM), MK801 (20 µM), SB-3CT (10 µM), and PAI-1 (5 nM) alone or in combination.

### SDS-PAGE gelatin and casein zymography

Gelatin zymograms were used to reveal matrix metalloproteinase-2 (MMP-2) and MMP-9 activities. Casein zymograms were used to reveal t-PA activity. Experiments were performed in P2 cortical extracts. Practically, microdissected cortices were washed in fresh aCSF and homogenized in 50 µL of lysis buffer (50 µM Hepes; pH 7.5; 150 mM NaCl; 10 mM EDTA; 10 mM glycerophosphate; 100 mM natrium fluoride; 1% Triton X-100). After centrifugation of the homogenates (20,000*g*× 15 min), the supernatants were collected and protein concentrations were determined by the Bradford assay. Proteins were loaded as 15 and 30 µg/well for gelatin and casein zymography, respectively, and separated by electrophoresis. For gelatin zymography, the gels were incubated in renaturing buffer (Triton X-100, 12.5 mL; H_2_O, 487.5 mL) for 30 min at room temperature and in developing buffer (glycine, 3.75 mM; EDTA, 1.45 mM) for another 30 min at room temperature. After transfer in fresh developing buffer, gels were incubated for 5 days at 37 °C, stained in 0.5% Coomassie blue for 1 h, and rinsed for 1 h in distilled water. Light stripes on a blue background indicated gelatin digestion of the gel by gelatinases. For casein zymography, gels were incubated in renaturing buffer at 4 °C overnight and rinsed in water for 30 min at 4 °C. Then, gels were transferred in developing buffer for 3 h and 30 min at 37 °C and stained with 0.5% Coomassie blue for 1 h. Finally, they were de-stained in 40% methanol and 10% acetic acid for 30 min at room temperature. Light stripes on a blue background indicated casein digestion of the gel by t-PA. Digital images of the gels were acquired and digestion stripes were quantified using Explora Nova Mercator Software (La Rochelle, France).

### In situ gelatinase and casein zymography on brain slices

In situ MMP and casein zymography were performed for 250-µm brain slices from wild-type (WT), t-PA^−/−^ or Grin1^lox/lox^/VeCad^Cre^ mice previously stained by isolectin-B4-TRITC to visualize the pial migratory route. Slices were incubated for 30 min in aCSF containing isolectin-B4-TRITC (1/50) at 37 °C in a humidified incubator under a controlled atmosphere of 5% CO_2_/95% air. After two washes for 10 min with aCSF, slices were incubated for 3 h with the quenched gelatinase substrate DQ-gelatin-FITC (1/50) or DQ-casein-FITC (1/125) in the presence of glutamate (100 µM), MK801 (20 µM), and/or SB-3CT (10 µM) alone or in combination. Quantification of FITC fluorescence, indicative of cell-specific digestion of the substrate, was performed by time-lapse recordings using a fluorescent videomicroscope system (DMI 6000B; Leica, Reuil-Malmaison, France) coupled to the Explora Nova Mercator Software. Microphotographs were acquired at 488 nm and 540 nm every 15 min for 3 h to follow the kinetics of DQ-gelatin-FITC and DQ-casein-FITC degradations and to visualize cortical microvessels from the pial migratory route, respectively. A background level was defined in a negative region of the slice.

### Laser capture microdissection and GluN1 qRT-PCR

Transverse slices (250 µm) were cut in ice-cold aCSF using a vibratome (VT1000S; Leica, Reuil-Malmaison, France) and stained with isolectin-B4-TRITC. Slices were transferred to FrameSlides with polyethylene terephthalate (PET, Steel frames, PET-membrane; Leica, Reuil-Malmaison, France), fixed in dry ice–cooled isopentane. Just before the LCM procedure, the samples were dehydrated for a few minutes with increasing concentrations of ethanol (70 and 100%). The laser capture microdissection was performed using a Leica Microdissection apparatus (LMD6000, Leica Microsystems), by applying a pulsed UVA laser beam (focal spot < 5 μm) through an inverted microscope with × 5–20 objectives (Supplementary Fig. 1a–e). After microdissection, vessel-enriched fractions were collected by gravity into a tube containing 60 μL of lysis buffer (Qiagen, Courtaboeuf, France) with 1% β-mercaptoethanol (Sigma-Aldrich). Total RNAs were extracted using an RNeasy Micro kit (Qiagen) in accordance with the manufacturer’s instructions and eluted with 14 μL of RNase-free water. DNase I treatment (RNase-Free DNase Set; Qiagen) was performed to remove contaminating genomic DNA. The quality of the RNAs was assessed (RIN between 5.2 and 6.9), and the quantity was estimated by microfluidic capillary electrophoresis (Agilent 2100 Bioanalyzer; Agilent Technologies, Massy, France). Approximately 20 ng of RNA were reverse-transcribed in a 20-mL reaction that contained 5× QuantiTec Buffer, RT Primer Mix and Quantiscript Reverse Transcriptase from a QuantiTec Reverse Transcription kit (Qiagen). Grin1 quantitative RT-PCR was performed using primers 5′-CTCTAGCCAGGTCTACGCTATCC (sense) and 5′-GACGGGATTCTGTAGAAGCCA (antisense). GAPDH was detected with primers 5′-CATGGCCTTCCGTGTTCCTA (sense) and 5′-CCTGCTTCACCACCTTCTTGA (antisense).

### Immunohistochemistry

Brain slices previously fixed with 4% PFA in PBS were incubated overnight at 4 °C with various primary antibodies diluted in incubation buffer (PBS containing 1% BSA and 3% Triton X-100) (Supplementary Table 1). Then, the slices were rinsed three times with PBS for 20 min and incubated with the same incubation buffer containing an adequate secondary antibody. Cell nuclei were visualized by incubating slices for 5 min with 1 µg/mL Hoechst 33258 in PBS. Fluorescent signals were observed with a Leica DMI 6000B microscope. The intensity profiles of immunolabeled slices were done using the *Line scan* tool of the Metamorph software (Roper Scientific, Evry, France). Control for nonspecific binding of the secondary antibody was performed by omitting the primary antibodies.

### Western blot

Microdissected cortices from WT and transgenic mice were rapidly homogenized in ice-cold lysis buffer. After centrifugation, supernatants were collected and protein concentrations were determined by the Bradford assay. The pellet was denatured at 100 °C for 5 min in 50 µL of Tris/HCl (pH 7.5) containing 20% glycerol, 0.7 M 2-mercaptoethanol, 0.004% (w/v) bromophenol blue, and 3% (w/v) sodium dodecyl sulfate (SDS), and then, it was electrophoresed on 10% SDS–polyacrylamide gel. After separation, the proteins were electrically transferred to a polyvinyldifluoridine membrane (PerkinElmer Life Sciences, Boston, MA). The membrane was incubated with blocking solution (5% BSA in Tris-buffered saline containing 0.05% Tween 20) at room temperature for 1 h and incubated overnight with primary antibodies against somatostatin and actin (Supplementary Table 1). After incubation with the corresponding secondary antibodies coupled to peroxidase (Santa Cruz Biotechnology, Santa Cruz, CA), proteins were visualized using an enhanced chemiluminescence ECL Plus immunoblotting detection system (ECL; Bio-Rad Laboratories, Marne la Coquette, France). The intensity of the immunoreactive bands was quantified using a blot analysis system (Bio-Rad Laboratories), and β-actin was used as a loading control. Commercial markers (Seeblue pre-stained standard; Invitrogen) were used as molecular weight standards.

### Quantification of the vessel-associated GABAergic interneurons and measurement of the PMR thickness

Quantification of vessel-associated GABAergic interneurons was performed after double immunostaining of P2 cortical slices with GABA and CD31 antibodies (Supplementary Table 1). Images were acquired and saved in TIFF format using a Leica TCS SP8 MP confocal microscope. Image resolution was 1024 × 1024 pixels and z stacks were done with a step ranging from 0.25 to 0.5 µm (Supplementary Fig. 2d). X/Z as well as Y/Z sections of the acquired stacks were done using the IMARIS imaging software (Bitplane, Zurich, Switzerland) to validate the vessel–neuron interactions (Supplementary Fig. 2a–c). Images were subsequently opened in Leica LAS AF Lite software to quantify the distance between the outer part of the neuron and the outer part of the vessel (Supplementary Fig. 2e). Distances below 15 µm were considered as representative of a vessel association (Supplementary Fig. 2e). Finally, the vessel density was quantified in the superficial and deep cortical layers, and this parameter was used to balance the vessel-associated vs not-associated proportion of GABA interneurons. The quantification of the PMR and cortical layer thicknesses in t-PA null mice and in Grin1^*lox/lox*^/VeCad^*Cre*^ mice was also performed using the Leica LAS AF Lite software.

### Quantification of GABA, Gad67, and somatostatin neuronal densities

For measurements of GABA neuronal density in the superficial cortical layers of wild type, t-PA^−/−^ and Grin1^flox^/VeCadherin^Cre^ mice, images were acquired at 20× magnification after different immulabeling using GABA, Gad67 or somatostatin antibodies. Practically, after a blind acquisition of the image at the tiff format, a background value was acquired using the Leica MM AF software powered by Metamorph V1.5. Then, the mean background value was subtracted using the “background correction” item. A region of interest (ROI) was defined within the superficial cortical layers and a segmentation done. The Metamorph analysis gave access to the number of objects (cell bodies) within the ROI and to the thresholded area/ROI area ratio. Morphometric analysis was then validated by the investigator to correct possible artefacts (for example, two fused cells counted as a single object or the presence of fluorescent debris). The analysis was repeated to cover the superficial layers I–IV from the somatosensory cortex in both hemispheres and in three slices per animal [29].

### Quantification of primary neurite density in double transgenic Gad67-GFP/t-PA^−/−^ mice

Quantification of primary neurite densities of GABA interneurons populating the cortical superficial layers was performed at P15 on both Gad67-GFP/t-PA^WT^ and Gad67-GFP/t-PA^−/−^ mice. A reference focal plane corresponding to the Gad67-GFP cell body was first defined with a confocal laser scanning microscope (Noran Instruments, Middleton, WI) using INTERVISION software (Noran Instruments). Then, Z-series of four images were acquired with a 2-µm step on both sides of the reference plane and saved in TIFF format. Next, acquired images were deconvolved and compiled for three-dimensional (3D) reconstruction using AutoQuant X_3_ deconvolution software (Media Cybernetics, Rockville, MD) to quantify the number of primary neurites per cell body.

### Quantification of spine morphology and density in Gad67-GFP/t-PA^−/−^ mice

A fine network of secondary and tertiary dendrites was acquired from Gad67-GFP/t-PA^WT^ and double transgenic Gad67-GFP/t-PA^−/−^ mice using a Leica TCS SP2 AOBS confocal laser scanning imaging system (Leica Microsystems AG). Z-stack series of images were later deconvolved using AutoQuant X_3_ software and loaded into IMARIS imaging software (Bitplane, Zurich, Switzerland) for 3D reconstruction. The Filament Tracer function was used to assign and classify spines, thus yielding spine density and spine categories (stubby, filopodia-shaped, or mushroom-shaped). For a given slice, two ROIs were defined, two-to-three dendrites were quantified per ROI, and the total number of quantified neuritis was fixed at 40 per group.

### Quantification of tangential and radial migration

Brain slices from P2 neonates were previously stained by isolectin-B4-TRITC to localize the vascular network of the pial migratory route. To label cortical cells, slices were incubated for 5 min in 10 µM Cell Tracker Green (CTG; Invitrogen) added to the culture medium. The chemically defined culture medium was composed of DMEM/Ham’s F12 with 1% N2 supplement and 1% antibiotic–antimycotic solution (Sigma-Aldrich). Slices were subsequently washed with culture medium, placed onto a polyester membrane insert from a 6-well plate, and incubated in a 5% CO_2_ atmosphere. After labeling, slices were transferred to an incubator attached to the stand of a confocal macroscope (TCS LSI; Leica Microsystems). The temperature of the chamber was kept at 37.0 ± 0.5 °C using a temperature controller (Tempcontrol 37-2 digital 2-channel; PeCon, Ulm, Germany), and the slices were supplied with a constant gas flow (95% O_2_, 5% CO_2_; CO_2_ controller; PeCon). To visualize cell migration in the tissue slices, the preparation was illuminated with a 488-nm wavelength light by means of a laser diode through a confocal laser scanning macroscope equipped with a × 2 dry objective (working distance, 39 mm; diameter, 58 mm; Leica Microsystems). Fluorescence emission was detected from 500 to 530 nm. To finely resolve the movement of interneurons, images were acquired with an additional optical zoom factor of 1.5–2.0. Images were collected every 30 min for up to 6 h. Tracking maps were designed using Metamorph and ImageJ (NIH) upgraded with the *tracking* plug-in allowing access to the total distance covered and maximal speed parameters [30].

### Quantification of locomotor activity of t-PA^−/−^ mice

Locomotor activity of P15 WT and t-PA^−/−^ mice was assessed using a Versamax 4.2 apparatus (Accuscan Instruments Inc., Columbus, OH, USA) and the behavioral analysis platform of the Institute of Research and Innovation in Biomedicine (IRIB; Normandy University). This motor behavioral test was chosen, because morphometric, mechanistic, and functional studies were performed in the sensorimotor prefrontal cortex, and previous studies reported that alteration of the GABAergic system in the prefrontal cortex is associated with locomotor activity impairments [31]. Animals were isolated for 30 min in individual cages before being placed in individual 20-cm × 20-cm × 30-cm compartments to limit anxiety in a dark, sound-attenuated, and temperature-regulated (20 ± 1 °C) room. The test takes advantage of a rodent’s natural tendency to explore a new environment. Video-tracking was performed during a 30-min period and analyzed using ANY-maze software (Stoelting, Wood Dale, IL, USA).

### In vivo exposure of Gad67-GFP mice to GM6001

The MMP inhibitor GM6001 (10 mg/kg) was administered daily by subcutaneous injection to pregnant Gad67-GFP mice from E15 to birth and in neonates from birth to P7. The dose of GM6001 administered was based on previous comparative studies that showed that a 10-mg/kg GM6001 subcutaneous injection significantly decreased cortical MMP activity [32]. The control group was treated with 1% DMSO in PBS. No modification of body weight of pregnant mice was seen in the control and GM6001-treated groups; however, the body weight of pups from the GM6001 group was significantly reduced. After birth, pups were raised until P15 to quantify the density of cortical Gad67-GFP interneurons.

### Statistical analysis

Statistical analyses were performed using the biostatistic Prism software (GraphPad Software, La Jolla, CA, USA). Tests used for each experiment, the number of independent experiments, the number of measures per experiment, and *p* values are summarized in Table 1. Error bars in the graphs represent SEM.

**Table 1:** Statistical analysis

Experiments	Test	*n* independents experiments	*p* value *p < 0.05; **p < 0.01; ***p < 0.001; ****p < 0.0001
Figure 1j Comparison of the vascular compartments in superficial and deep cortical layers	Unpaired *t* test	Seven slices from three independent animals *n* = 3	*p* = 0.7802, ns
Figure 1j Distribution of vessel-associated GABA interneurons	Chi^2^	Seven slices from three independent animal *n* = 3 s	Chi^2^, *df* 11.75, 1 *p* = 0.0006***
Figure 2h gel zymography dose effect of glutamate on MMP9 activity	One-way ANOVA Dunnett post-test	*n* = 3 independent experiments	ANOVA F 4.733 *p* = 0.0350* Dunnett’s *p* = 0.0162*
Figure 2i Gel zymography effect of MK801 on glutamate-induced MMP9 activity	One-way ANOVA Tukey post-test	*n* = 3 independent experiments	ANOVA F 15.23 *p* = 0.0011** Tukey’s Ctrl vs Glut *p* = 0.0114* Glut vs MK801 *p* = 0.0012^##^ Glut vs Glut + MK *p* = 0.0028^##^
Figure 2j Gel zymography effect of SB3CT on glutamate-induced MMP9 activity	One-way ANOVA Tukey post-test	*n* = 3 independent experiments	ANOVA F 30.72 *p* < 0.0001**** Tukey’s Ctrl vs Glut *p* = 0.0014** Glut vs SB3CT *p* < 0.0001^####^ Glut vs Glut + SB3CT *p* = 0.0009^###^
Figure 3h In situ zymography effect of glutamate and MK801 on MMPs activity on P2 slices	Two-way ANOVA Tukey post-test	*n* = 5 slices from different animals	ANOVA Interaction F 2.793 *p* < 0.0001**** Treatment F 48.68 *p* < 0.0001**** Time F 137.8 *p* < 0.0001**** Tukey’s For *t* = 180 min Ctrl vs GLUT *p* < 0.0001**** Glut vs Glut + MK801 *p* < 0.0001^####^ For *t* = 165 min Ctrl vs GLUT *p* < 0.0001**** Glut vs Glut + MK801 *p* < 0.0001^####^
Figure 3i In situ zymography effect of glutamate and SB3CT on MMPs activity on P2 slices	Two-way ANOVA Tukey post-test	*n* = 5 slices from different animals	ANOVA Interaction F 1.538 *p* = 0.0339* Treatment F 30.25 *p* < 0.0001**** Time F 57.17 *p* < 0.0001**** Tukey’s For *t* = 180 min Ctrl vs Glut *p* < 0.0001**** Glut vs Glut + SB3CT *p* < 0.0001^####^ For *t* = 165 min Ctrl vs GLUT *p* = 0.0042** Glut vs Glut + SB3CT *p* < 0.0001^####^
Figure 4b Gel zymography effect of MK801 on glutamate-induced tPA activity	One-way ANOVA Tukey post-test	*n* = 3	ANOVA F 7.638 *p* = 0.0014** Tukey’s Ctrl vs Glut *p* = 0.0079** Glut vs MK801 *p* = 0.0011^##^ Glut vs Glut + MK801 *p* = 0.0495^#^
Figure 4c Gel zymography effect of PAI-1 on glutamate-induced tPA activity	One-way ANOVA Tukey post-test	*n* = 3	ANOVA F 5.610 *p* = 0.0059** Tukey’s Glut vs PAI *p* = 0.0043^##^ Glut vs Glut + MK *p* = 0.0365^#^
Figure 4g In situ zymography effect of glutamate on DQ-casein activity	Two-way ANOVA Tukey post-test	*n* = 5 slices from different animals	ANOVA Interaction F 3.329 *p* < 0.0001**** Treatment F 33.70 *p* < 0.0001**** Time F 286.2 *p* < 0.0001**** Tukey’s For *t* = 180 min Ctrl vs Glut *p* < 0.0001**** Glut vs Glu + MK801 *p* < 0.0001^####^ For *t* = 165 min Ctrl vs GLUT *p* < 0.0001**** Glut vs Glut + MK801 *p* < 0.0001^####^
Figure 4h Gel zymography effect of glutamate on MMP9 activity in tPA^−/−^ mice	Unpaired *t* test	*n* = 3	WT *p* = 0.0014** tPA^−/−^*p* = 0.2720, ns
Figure 4i In situ zymography effect of glutamate on MMPs activity in tPA^−/−^	Two-way ANOVA Tukey post-test	*n* = 4	ANOVA Interaction F 0.6479 *p* = 0.9364, ns Treatment F 3.818 *p* < 0.0001**** Time F 32.09 *p* < 0.0001**** Tukey’s For *t* = 180 min Ctrl WT vs Glut WT *p* = 0.0008*** Ctrl tPA^−/−^ vs Glu tPA^−/−^ *p* = 0.9898, ns For* t* = 165 min Ctrl WT vs Glut WT *p* = 0.005** Ctrl tPA^−/−^ vs Glu tPA^−/−^ *p* = 0.9982, ns
Figure 5g Time-lapse videomicroscopy migration speed	One-way ANOVA Tukey post-test	*n* = 3 at least 20 cells tracked from three slices from three different animals per group Normality controlled using the D’Agostino & Pearson normality test	Radial ANOVA F 33.5 *p* = 0.0006*** Tukey’s Ctrl vs Glut *p* = 0.0052** Glut vs MK *p* = 0.0005^###^ Tangential ANOVA F 12.2 *p* = 0.0077** Tukey’s Ctrl vs Glut *p* = 0.024* Glut vs MK *p* = 0.008^##^
Figure 6e PMR thickness and distance from PMR in tPA-null mice	Unpaired *t* test	*n* = 3 at least two slices per animal Three independent animals studied	Thickness *p* = 0.0033** Distance from PMR *p* = 0.0002***
Figure 6h spine density in Gad67GFP/tPA^−/−^ mice	Unpaired *t* test	*n* = 3 *n* = 20 fibers from three animals	WT vs tPA^−/−^ males *p* = 0.4106, ns
Figure 6i Spine distribution in Gad67GFP/tPA^−/−^	Chi-square test	*n* = 20 fibers analyzed from three animals	Chi-square, *df* 6.329, 2 *p* = 0.422*
Figure 6j Quantification of motor activity in tPA^−/−^ mice	Unpaired *t* test	Males *n* = 8 Females *n* = 8 Males + females *n* = 16	WT vs tPA^−/−^ males *p* = 0.0211* WT vs tPA^−/−^ frmales *p* = 0.027* WT vs tPA^−/−^ females + males *p* = 0.0032**
Figure 6m Effect of GM6001 on the density of Gad67GFP cells at P15	Unpaired *t* test	Males *n* = 4 Females *n* = 4 Males + females *n* = 8	Ctrl vs GM6001 males *p* = 0.004** Ctrl vs GM6001 females *p* = 0.1420, ns Ctrl vs males +females *p* = 0.0006***
Figure 6n Effect of GM6001 on the distribution of Gad67GFP cells between superficial and deep cortical layers at P15	Chi-square test	5 ROI were analyzed per slice from three animals	Chi-square, *df* 14.71, 1 *p* = 0.0001****
Figure 7d Thickness of cortical layers in Grin1^lox/lox^/VeCad^Cre^ mice at P0	One-way ANOVA Tukey’s post-test	*n* = 3 at least 10 ROI analyzed from three independent animals	PMR ANOVA F 139.7 *p* < 0.0001**** Tukey’s multiple comparison test WT vs Grin1^lox/lox^/VeCad^cre^*p* < 0.0001**** Grin1^lox/lox^/VeCad^+/+^ vs Grin1^lox/lox^/VeCad^cre^ *p* < 0.0001^####^ Layers I–IV ANOVA F 14.55 *p* < 0.0001**** Tukey’s multiple comparison test WT vs Grin1^lox/lox^/VeCad^cre^*p* = 0.001** Grin1^lox/lox^/VeCad^+/+^ vs Grin1^lox/lox^/VeCad^cre^ *p* < 0.0001^####^ Layers V–VI ANOVA F 4.213 *p* = 0.026* Tukey’s multiple comparison test WT vs Grin1^flox^/VeCad^cre^*p* = 0.1061 ns Grin1^lox/lox^/VeCad^+/+^ vs Grin1^lox/lox^/VeCad^cre^ *p* = 0.0269^#^
Figure 7e Cortical densities of GABA interneurons Grin1^lox/lox^/VeCad^Cre^ mice at P0	Two-way ANOVA Tukey’s post-test	*n* = 3 at least 4 ROI analyzed from three independent animals	ANOVA Interaction Genotype F 32.39, *p* < 0.0001**** Interaction Layers F 5.87, *p* < 0.0001**** Tukey’s post-test PMR WT vs Grin1^lox/lox^/VeCad^cre^ *p* < 0.0001**** Grin1^lox/lox^/VeCad^+/+^ vs Grin1^lox/lox^/VeCad^cre^ *p* < 0.0001^####^ I–IV WT vs Grin1^lox/lox^/VeCad^cre^ *p* = 0.0076** Grin1^lox/lox^/VeCad^+/+^ vs Grin1^lox/lox^/VeCad^cre^ *p* = 0.0201^#^ V–VI WT vs Grin1^lox/lox^/VeCad^cre^ *p* = 0.1785 ns Grin1^lox/lox^/VeCad^+/+^ vs Grin1^lox/lox^/VeCad^cre^ *p* = 0.0916 ns
Figure 7f In situ zymography effect of glutamate on MMPs activity in Grin1^lox/lox^/VeCad^Cre^ mice at P0	One-way ANOVA Tukey’s post-test	*n* = 3 slices from different animals	ANOVA F 8.309 *p* = 0.0187* Tukey’s WT vs Grin1^lox/lox^/VeCad^cre^*p* = 0.0216* Grin1^lox/lox^/VeCad^+/+^ vs Grin1^lox/lox^/VeCad^cre^ *p* = 0.0416^#^
Figure 7j Thickness of cortical layers in adult Grin1^lox/lox^/VeCad^Cre^ mice	Two-way ANOVA Tukey’s post-test	*n* = 3 at least 10 ROI analyzed from three independent animals	ANOVA Interaction Genotype F 14.64, *p* = 0.0002*** Interaction Layers F 2604, *p* < 0.0001**** Tukey’s post-test Layer I WT vs Grin1^lox/lox^/VeCad^cre^*p* = 0.0106* Grin1^lox/lox^/VeCad^+/+^ vs Grin1^lox/lox^/VeCad^cre^ *p* = 0.0056^##^ Layers II–IV WT vs Grin1^lox/lox^/VeCad^cre^*p* = 0.0003*** Grin1^lox/lox^/VeCad^+/+^ vs Grin1^lox/lox^/VeCad^cre^ *p* < 0.0001^####^ Layers V–VI WT vs Grin1^flox^/VeCad^cre^ *p* = 0.2113 ns Grin1^lox/lox^/VeCad^+/+^ vs Grin1^lox/lox^/VeCad^cre^ *p* = 0.3167 ns
Figure 7k Cortical densities of GABA interneurons in adult Grin1^flox^/VeCad^cre^ mice	Two-way ANOVA Tukey’s post-test	*n* = 3 at least 4 ROI analyzed from three independent animals	ANOVA Interaction Genotype F 16.79, *p* < 0.0001**** Interaction Layers F 50.16, *p* < 0.0001**** Tukey’s post- test Layer I WT vs Grin1^lox/lox^/VeCad^cre^*p* = 0.9662 ns Grin1^lox/lox^/VeCad^+/+^ vs Grin1^lox/lox^/VeCad^cre^ *p* = 0.6581 ns Layers II–IV WT vs Grin1^lox/lox^/VeCad^cre^*p* < 0.0001**** Grin1^lox/lox^/VeCad^+/+^*vs* Grin1^lox/lox^/VeCad^cre^ *p* < 0.0001^####^ Layers V–VI WT vs Grin1^flox^/VeCad^cre^ *p* = 0.5164 ns Grin1^lox/lox^/VeCad^+/+^ vs Grin1^lox/lox^/VeCad^cre^ *p* = 0.0964 ns
Suppl. Fig. 1f GluN1 Q-RTPCR on microdissected vessels	One-way ANOVA Tukey post-test	*n* = 3 three fractions collected per slice and repeated in three independent slices (from independent neonates)	ANOVA F 0.2789 *p* = 0.7659 ns Tukey’s Pial vs Subpial *p* = 0.7819 ns Pial vs Deep *p* = 0.9966 ns
Suppl. Fig. 5b Vessel orientation in t-PA^−/−^	Chi-square test	Seven slices from three animals	Chi-square, *df* 2.195, 3 *p* = 0.5329 ns
Suppl. Fig. 7i Western blot somatostatin in tPA^−/−^ (Suppl Fig. 3j)	Unpaired *t* test	Males *n* = 3 Females *n* = 3 Males + females *n* = 6	WT vs tPA^−/−^ males *p* = 0.0219* WT vs tPA^−/−^ females *p* = 0.055, ns WT vs tPA^−/−^ females + males *p* = 0.0753, ns
Suppl. Fig. 7j Primary dendrite density in tPA^−/−^ mice	Unpaired *t* test	Males *n* = 3 Females *n* = 3 Males + females *n* = 6	WT vs tPA^−/−^ males *p* = 0.2302, ns WT vs tPA^−/−^ females *p* = 0.2573, ns WT vs tPA^−/−^ females + males *p* = 0.0402*
Suppl. Fig. 7k Density of somatostatin cells in tPA^−/−^ mice (Suppl Fig. 3l)	Unpaired *t* test	Males *n* = 8 Females *n* = 8 Males + females *n* = 16	WT vs tPA^−/−^ males *p* = 0.0755, ns WT vs tPA^−/−^ females *p* = 0.0021** WT vs tPA^−/−^ females + males *p* = 0.001***

The tests used, the number of independent experiments, the number of measures per experiment, and *p* values are detailed for each experiment. Error bars in the graphs represent SEM

## Results

### Immunohistochemical characterization of the pial migratory route in neonate mice

**Fig. 1:**
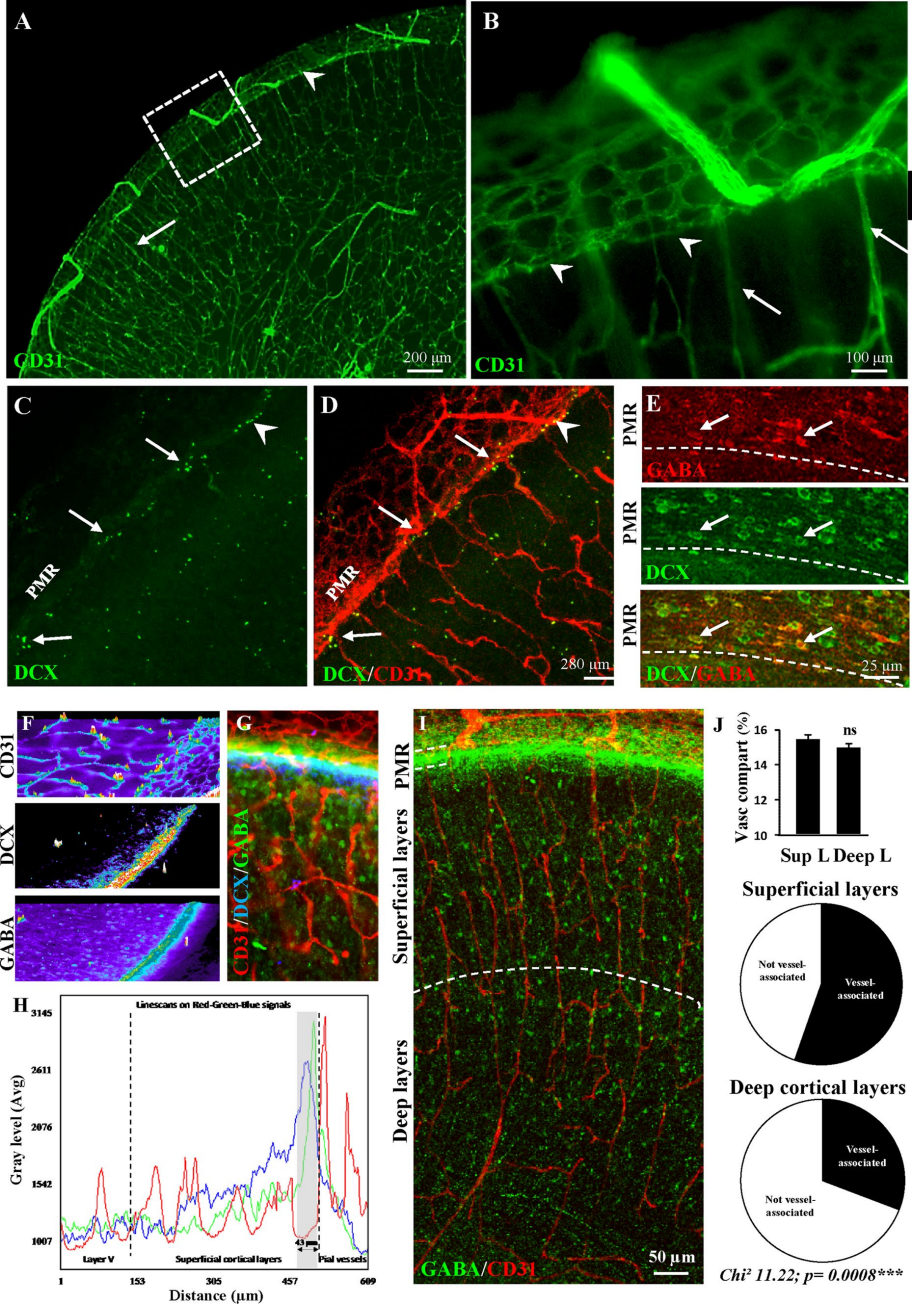
Immunohistochemical characterization of the pial migratory route (PMR) in mouse neonates. **a**, **b** Low-magnification (**a**) and high-magnification (**b**) photographs visualizing cortical microvessels in transversal cortical slices labeled with CD31 antibodies at P2. Arrow heads indicate a thin network of vessels at the level of the PMR. Arrows indicate cortical radial microvessels. **c**, **d** Double immunolabeling experiments showing a low-magnification DCX-positive cells (**c**) lining the PMR (**d**; arrowhead). Arrows indicate DCX-positive cells stacking at the level of radial microvessels arising from the PMR. **e** Double immunolabeling experiments showing at high-magnification tangential cells along the PMR immunoreactive for DCX and GABA (arrows). **f**–**h** Line scan analysis (**f**) of the CD31 (red), DCX (blue) and GABA (green) fluorescent signals acquired from a cultured brain slice at P2 (**g**). Intensity profiles (**h**) indicate that DCX and GABA immunofluorescences overlap and border the inner part of pial vessels. **i** Confocal acquisition of GABA-immunoreactive cells and CD31-labeled microvessels in the developing neocortex at P2. Note a marked vascular interaction of GABA interneurons along radial microvessels. **j** Quantification of vessel density (upper panel) and vessel-associated GABAergic interneurons in the superficial (middle) and deep (lower panel) cortical layers at P2. Note that the vessel association is preferentially observed in the developing superficial layers. *ns* not statistically different vs superficial layers. The tests used for the statistical analysis, the number of independent experiments, the number of measures per experiment, and *p* values are detailed in Table 1

Several research groups reported that a significant number of GABA interneurons are still migrating in the superficial cortical layers at birth and during the early postnatal life of both mouse [9] and human [18] neonates. However, most previous studies referring to vessel-associated migration were performed during embryonic stages [11, 33]. Based on these statements, we performed immunohistochemistry experiments on the pial migratory route in P2 neonate mice (Fig. 1). CD31 immunolabeling revealed a thin network of microvessels lining the developing superficial cortical layers (Fig. 1a, b; arrowheads) and radial perforating microvessels (Fig. 1a, b; arrows). Contrasting to the adult cortex used as positive control, immunohistochemistry experiments revealed an absence of Acta2-positive vessels in the developing cortex at P2, suggesting that the arterial/venous phenotype of radial vessels was not yet fully established at this developmental stage (Supplementary Fig. 3a–e; [61]). Co-labeling experiments using CD31 and GFAP antibodies revealed a row of astrocytes with radial-oriented short processes entering the superficial cortical layers (Supplementary Fig. 4a–c; arrows). DCX immunohistochemistry also showed tangentially oriented cells lining the inner face of the pial migratory route (Supplementary Fig. 4d–f; arrows). At low magnification, confocal acquisitions confirmed the presence of DCX-positive cells lining the PMR and revealed cell clusters stacking at the junction between the PMR and radial cortical microvessels (Fig. 1c, d; arrows). Co-labeled DCX-GABA cells with a tangential orientation were observed along the PMR (Fig. 1e) and a line scan analysis of triple-labeled DCX-GABA-CD31 cortical slices showed that both DCX and GABA-immunoreactive profiles overlapped and were localized just below pial vessels (Fig. 1f–h). In the same way, triple immunolabeling targeting CD31, GFAP, and DCX showed that DCX-positive cells were localized just below GFAP-positive cells and pial vessels (Supplementary Fig. 4g–l). In addition, confocal acquisitions at low magnification of double immunolabeling experiments targeting GABA and CD31 clearly showed a preferential while not exclusive association of GABA interneurons with radial microvessels in the superficial cortical layers (Fig. 1i; Supplementary Figs. 2a, c, 3f). Quantitative analysis revealed different “vessel-association” patterns between superficial and deep cortical layers at P2. Indeed, whereas the vessel density was similar between deep and superficial cortical layers, the proportion of interneurons interacting with microvessels appeared significantly more important in the superficial layers of the developing cortex (Fig. 1j). These data indicate that in mouse neonates, late migrating GABA interneurons are interacting with vessels from the pial migratory route and radial microvessels from the superficial cortical layers.

### NMDA receptors impact MMP-9 activity in the pial migratory route

**Fig. 2:**
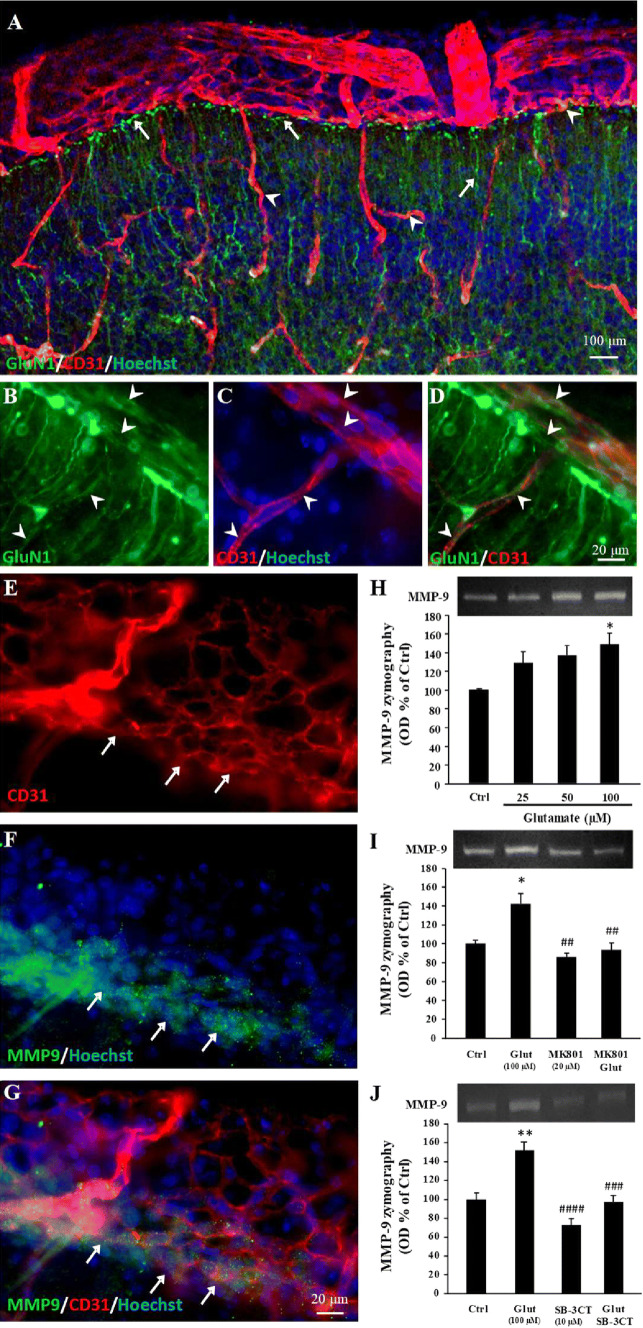
GluN1 immunoreactivity in microvessels from the pial migratory route (PMR) and glutamate-dependent regulation of MMP-9 activity. **a**–**d** Double immunolabeling experiments showing CD31-positive microvessels and GluN1 immunoreactivity in the neocortex of P2 mice. Immunohistochemistry reveals numerous GluN1-positive cortical radial fibers (**a**; arrows) and GluN1-positive endothelial cells in the pial migratory route (**b**–**d**; arrowheads). **e**–**g** Double immunolabeling experiments showing CD31-positive microvessels (**e**) and MMP-9 immunoreactivity (**f**) in vessels from the pial migratory route. The overlay (**g**) indicates that MMP-9 immunoreactivity mainly co-localizes with endothelial cells present in the inner part of the migratory route (arrows). **h** Quantification by gel zymography of the effects of 6-h exposure of P2 cortical slices to graded concentrations (25–100 µM) of glutamate on MMP-9 activity. **i** Quantification by gel zymography of the effect of the NMDA-antagonist MK801 (20 µM) on the glutamate-induced increase of MMP-9 activity. **j** Quantification by gel zymography of the effect of the MMP-9 inhibitor SB-3CT (10 µM) on the glutamate-induced increase of MMP-9 activity. *p < 0.05; **p < 0.01 vs control and ^##^*p* < 0.01; ^###^p < 0.001; ^####^p < 0.0001 vs glutamate. The tests used for the statistical analysis, the number of independent experiments, the number of measures per experiment, and *p* values are detailed in Table 1

Recent studies showed transcriptomic and proteomic specificities of cortical microvessels in neonate mice [1]. In particular, the expression of NMDA receptors is exacerbated in neonatal endothelial cells when compared to adults [6]. However, the distribution pattern of endothelial NMDA receptors in the developing cortex and the possible interactions with the pial migratory route was unknown. GluN1 immunohistochemistry experiments performed on P2 cortices showed intense immunolabeling in radial glia with numerous terminal processes ending at the level of the pial migratory route (Fig. 2a; arrows). GluN1 immunoreactivity was also detected in endothelial cells from the pial migratory route as well as in radial microvessels (Fig. 2b–d; arrowheads). Quantification of the GluN1 mRNA expression by qRT-PCR after laser capture revealed no significant difference between the pial, subpial, and deep microdissected microvessels (Supplementary Fig. 1f). Although MMP-9 is well known to contribute to the pathophysiology of several diseases involving dysfunction of the neurovascular unit during stroke [34] or dysregulation of cell migration with cancers [35], the contribution of MMP-9 to the control of vasculo-associated neuronal migration has never been investigated. Immunohistochemistry showed strong MMP-9 labeling at the level of the pial migratory route (Fig. 2e–g). Interestingly, MMP-9 immunolabeling was preferentially localized in the inner face of the pial vessels where migrating GABA interneurons are observed (Fig. 2e–g; arrows). Gel zymography experiments performed with P2 cultured cortical extracts showed that glutamate induces a concentration-dependent increase of MMP-9 activity (*p* < 0.05; Fig. 2h). The glutamate-induced increase of MMP-9 activity was abolished by the NMDA receptor antagonist MK801 (20 µM; *p* < 0.01; Fig. 2i) and by the MMP-9 inhibitor SB-3CT (10 µM; *p* < 0.001; Fig. 2j).

**Fig. 3:**
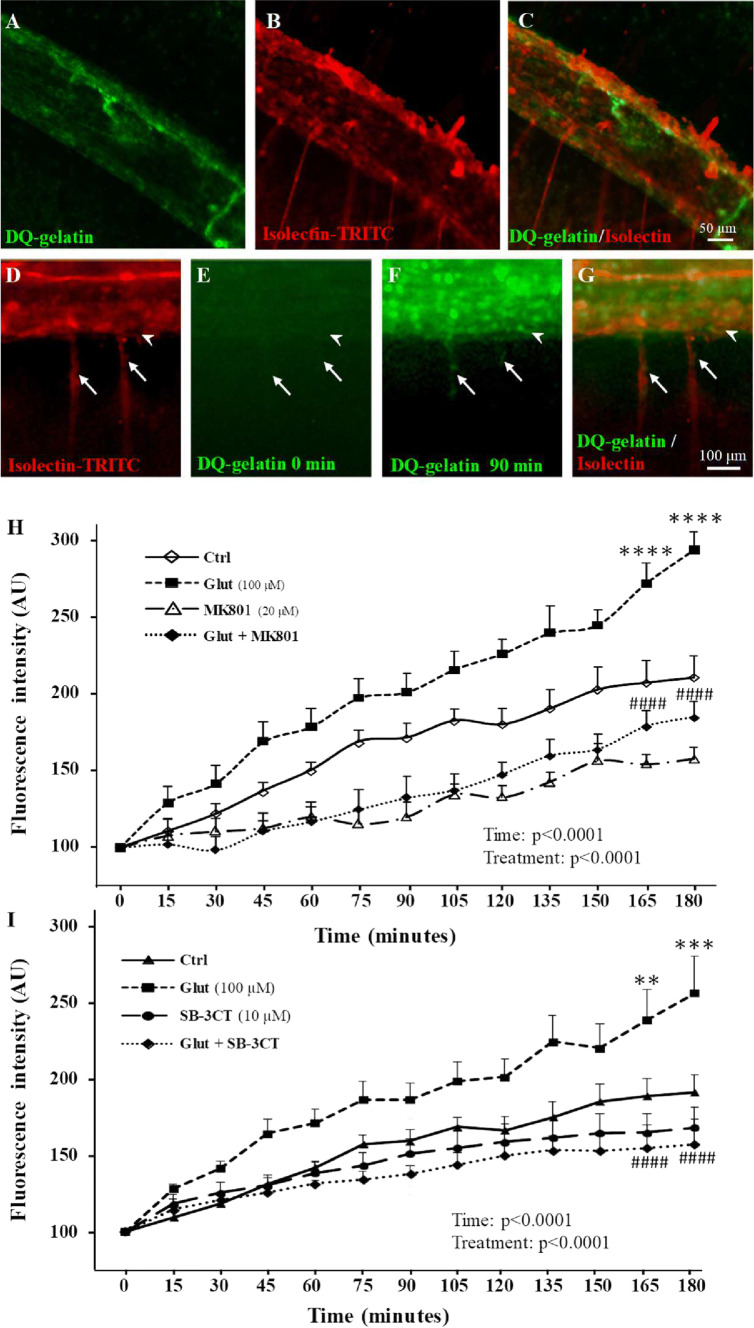
In situ characterization of the effects of glutamate on MMP activity in vessels from the pial migratory route. **a**–**c** In situ zymography performed for P2 cortical slices visualizing the MMP gelatinase activity in control conditions after 60-min incubation with the quenched fluorogenic substrate DQ-gelatin-FITC. Note that the gelatinase activity (**a**) overlaps with microvessels present in the pial migratory route labeled with isolectin-TRITC (**b**, **c**). **d**–**g** Microphotographs acquired by videomicroscopy show a time-dependent increase of the gelatinase activity in cultured slices incubated with glutamate (100 µM) from 0 to 90 min. Note that in addition to the PMR (arrowheads), a fluorescent signal also progressively appeared in radial cortical vessels (arrows; **f**, **g**). **h** Time-lapse quantification by videomicroscopy of the fluorescence intensity resulting from DQ-gelatin-FITC cleavage after treatment of brain slices with aCSF (Ctrl), glutamate (100 µM), and the NMDA-antagonist MK801 (20 µM) alone or co-incubated (**h**). **i** Quantification by videomicroscopy of the fluorescence intensity resulting from DQ-gelatin-FITC cleavage after treatment of brain slices with aCSF (Ctrl), glutamate (100 µM), and the MMP2/9 inhibitor SB-3CT (10 µM) alone or co-incubated. Statistical analysis revealed significant Time and Treatment interactions. **p < 0.01; ***p < 0.001; ****p < 0.0001 vs control at the same time point and ^####^*p* < 0.0001 vs glutamate at the same time point. The tests used for the statistical analysis, the number of independent experiments, the number of measures per experiment, and *p* values are detailed in Table 1

Because gel zymography performed on P2 cortical extracts provided data regarding MMP-9 activity but gave no information regarding histological localization, we also performed real time in situ zymography for cultured P2 cortical slices using DQ-gelatin-FITC (Fig. 3a–g). Co-labeling of cortical microvessels with isolectin-B4-TRITC revealed that gelatinase activity was predominantly vascular and detected in the pial vessels (Fig. 3a–c). Time-lapse acquisition also showed gelatinase activity in radial microvessels restricted to the superficial cortical layers (Fig. 3f, g; arrows). Quantification of the gelatinase activity showed that glutamate (100 µM) induces significant (*p* < 0.0001) and time-dependent increases of DQ-gelatin-FITC proteolysis (Fig. 3h). This effect was abrogated by MK801 (20 µM; Fig. 3h) as well as by SB-3CT (10 µM; Fig. 3i). These data indicate that in P2 cortical slices, glutamate is able to stimulate MMP-9 activity in vessels from the pial migratory route through an NMDA receptor-dependent mechanism.

### Glutamate controls t-PA activity in the pial migratory route

**Fig. 4:**
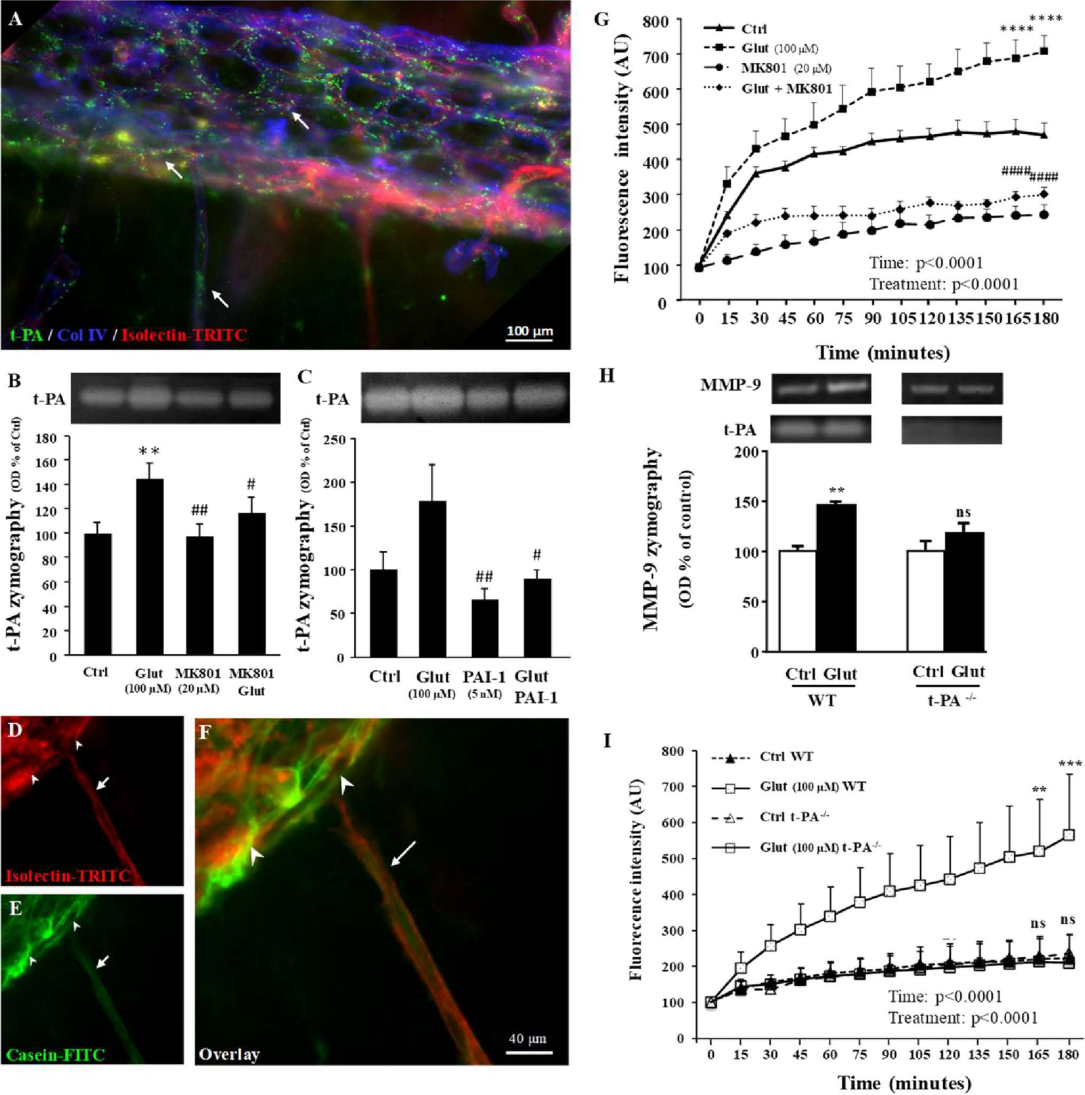
Visualization and quantification of the effects of glutamate on t-PA activity in vessels from the pial migratory route. **a** Triple labeling visualizing t-PA, collagen IV, and microvessels in the pial migratory route. Note the presence of strong vascular t-PA immunoreactivity in both tangential and radial cortical vessels (arrows). **b** Quantification by gel zymography of the effects of aCSF (Ctrl), glutamate (100 µM), and the NMDA-antagonist MK801 (20 µM) alone or co-incubated on t-PA activity after 3-h treatment of P2 cortical slices. **c** Quantification by gel zymography of the effects of aCSF (Ctrl), glutamate (100 µM), and the t-PA inhibitor PAI-1 (5 µg/mL) alone or co-incubated on t-PA activity after 3-h treatment of P2 cortical slices. **d**–**f** Microphotographs acquired by in situ zymography and visualizing cortical microvessels (**d**) and the fluorescent signal resulting from the cleavage of the quenched casein-FITC substrate (**e**) in cultured P2 cortical slices. Overlay reveals a strong protease activity associated with the pial migratory route (arrowheads) and radial cortical microvessels (arrows, **f**). **g** Quantification by time-lapse videomicroscopy of the fluorescence intensity resulting in casein-FITC cleavage after incubation of P2 brain slices with aCSF (Ctrl), glutamate (100 µM), and MK801 (20 µM) alone or co-incubated. **h** Quantification by gel zymography of the effects of glutamate (100 µM) on MMP-9 activity in P2 cortical extracts from wild-type (WT) and t-PA^−/−^ mice. **i** Quantification by in situ zymography of the effects of glutamate (100 µM) on the cleavage of the quenched casein-FITC substrate in WT and t-PA^−/−^ mice at the level of the pial migratory route in P2 cortical slices. Statistical analysis revealed significant Time and Treatment interactions. *ns* not significant; **p < 0.01; ***p < 0.001; ****p < 0.0001 vs control and ^####^*p* < 0.0001 vs glutamate. The tests used for the statistical analysis, the number of independent experiments, the number of cells tracked per experiment, and *p* values are detailed in Table 1

Previous studies have reported that t-PA is able to promote MMP-9 activation in a pathological context [36], whereas other research groups mentioned delayed neuronal migration in t-PA^−/−^ mice [37]. Considering this literature, it was tempting to speculate the contribution of t-PA to the control of MMP-9 activity in the pial migratory route. Immunohistochemistry experiments revealed intense t-PA labeling in both the pial and radial microvessels entering the superficial cortical layers (Fig. 4a; arrows). In addition, gel zymography experiments indicated that 6-h incubation of cultured P2 cortical slices with glutamate (100 µM) induces a significant increase in t-PA activity (*p* < 0.01; Fig. 4b). This effect was blocked by MK801 (20 µM; *p* < 0.05; Fig. 4b) and the t-PA inhibitor PAI-1 (5 nM; *p* < 0.05; Fig. 4c). Real-time in situ zymography performed on P2 cortical slices using DQ-casein-FITC showed that protease activity was localized in vessels bordering the pial migratory route (arrowheads) as well as in cortical radial microvessels (Fig. 4d–f; arrows). Quantification indicated that glutamate (100 µM) induced a time-dependent and significant increase of the DQ-casein-FITC proteolysis (*p* < 0.0001; Fig. 4g). This effect of glutamate was abrogated by MK801 (*p* < 0.0001; Fig. 4g). In addition, gel and in situ zymography revealed that treatment of P2 cortical slices from t-PA^−/−^ mice with glutamate (100 µM) failed to stimulate MMP-9 activity (Fig. 4h, i; Supplementary Fig. 5c, h). These data indicate that glutamate is able to stimulate t-PA activity in vessels from the pial migratory route through a NMDA receptor-dependent mechanism and that glutamate-induced MMP-9 activation is t-PA-dependent.

### Glutamate controls vascular-associated migration of GABA interneurons in the pial migratory route

**Fig. 5:**
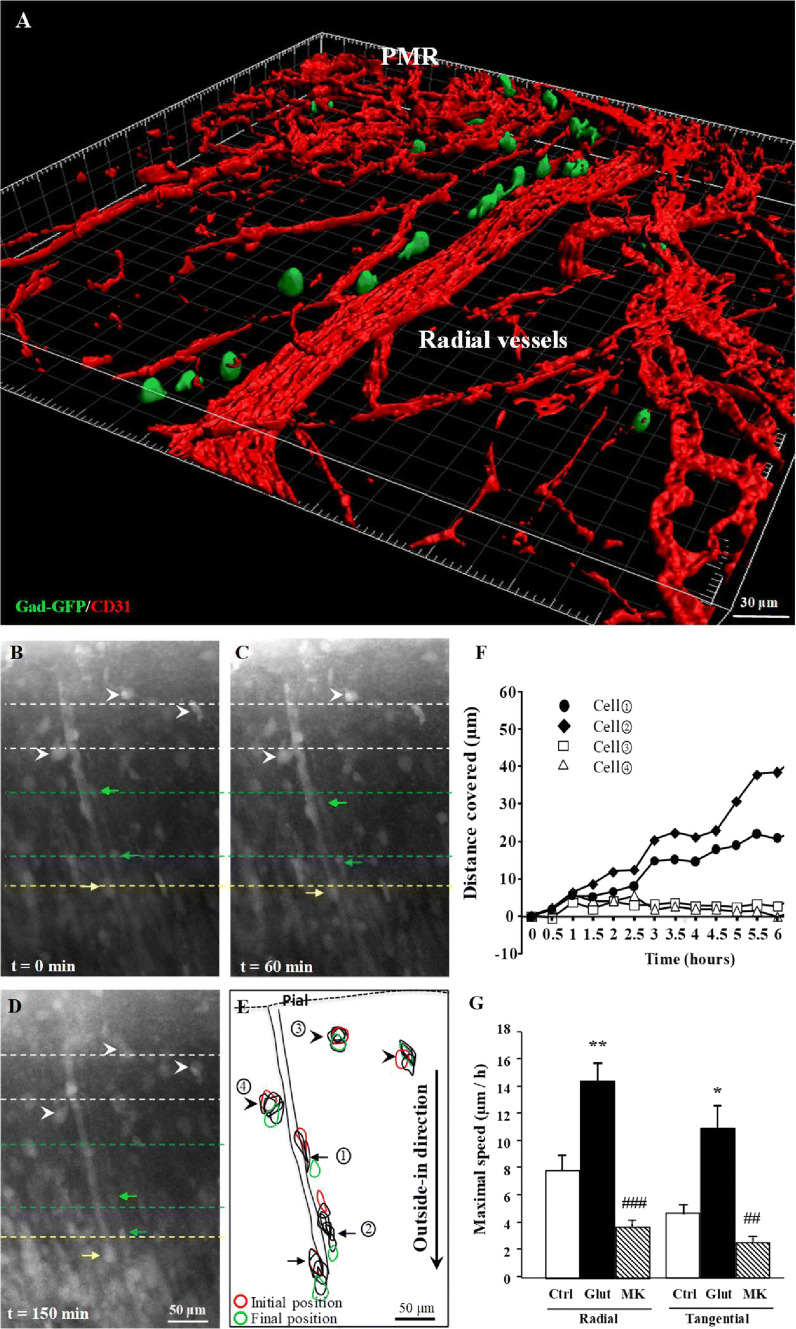
Effects of glutamate and MK801 on the vessel-associated migration of GABA interneurons. **a** 3D Imaris modeling of migrating interneurons along radial microvessels in Gad67-GFP mice at P2. GFP-positive neurons were visualized by immunohistochemistry and microvessels labeled with CD31 antibodies. **b**–**d** Microphotographs acquired from the same cultured slice at different times of tracking (0 min, **b**; 60 min, **c**; and 150 min, **d**). Arrows indicate migrating cells along microvessels. Arrowheads indicate immobile large cells. **e** Stacked graph visualizing the position of several cells during 6-h video-tracking. **f** Quantification of the distance covered by the four cells identified in **e**. **g** Effects of glutamate (50 µM) and MK801 (20 µM) on the migration speed of cells moving tangentially and radially along microvessels. **p < 0.01 vs control and ^###^*p* < 0.001 vs glutamate. The tests used for the statistical analysis, the number of independent experiments, the number of measures per experiment, and *p* values are detailed in Table 1

Because t-PA and MMP-9 are involved in the remodeling of the extracellular matrix following ischemic stroke [38], and because the present study revealed NMDA-dependent effects of glutamate on the endothelial activity of these two proteases, we hypothesized that glutamate could regulate the vascular-associated migration of GABA interneurons along the pial migratory route. Gad67-GFP mice whose neurons are known to populate the superficial cortical layers (Supplementary Fig. 6; [25]) confirmed the preferential association with microvessels at P2 (Fig. 5a). To investigate the effects of glutamate on the migration of GABAergic cells along the PMR and radial vessels, we performed time-lapse acquisitions using a confocal laser scanning macroscope (Fig. 5b–g). Time-lapse acquisitions revealed several cells migrating in an outside-in direction in close interaction with microvessels (Fig. 5b–e; arrows; Supplementary video). Some cells close to microvessels covered up to 40 µm of distances during the first 6 h of recording (Fig. 5f). The addition of glutamate (50 µM) to the culture medium significantly increased the maximal speed of both tangential and radial migrating cells (*p* < 0.01; Fig. 5g). In contrast, incubation of the slices with MK801 (20 µM) reduced cell migration (*p* < 0.001; Fig. 5g). These data indicate that in neonates, glutamate stimulates the vascular-associated migration of GABA interneurons through a NMDA receptor-dependent mechanism.

### t-PA invalidation and in vivo MMP inhibition impair the integration of GABA interneurons migrating via the pial migratory route

**Fig. 6:**
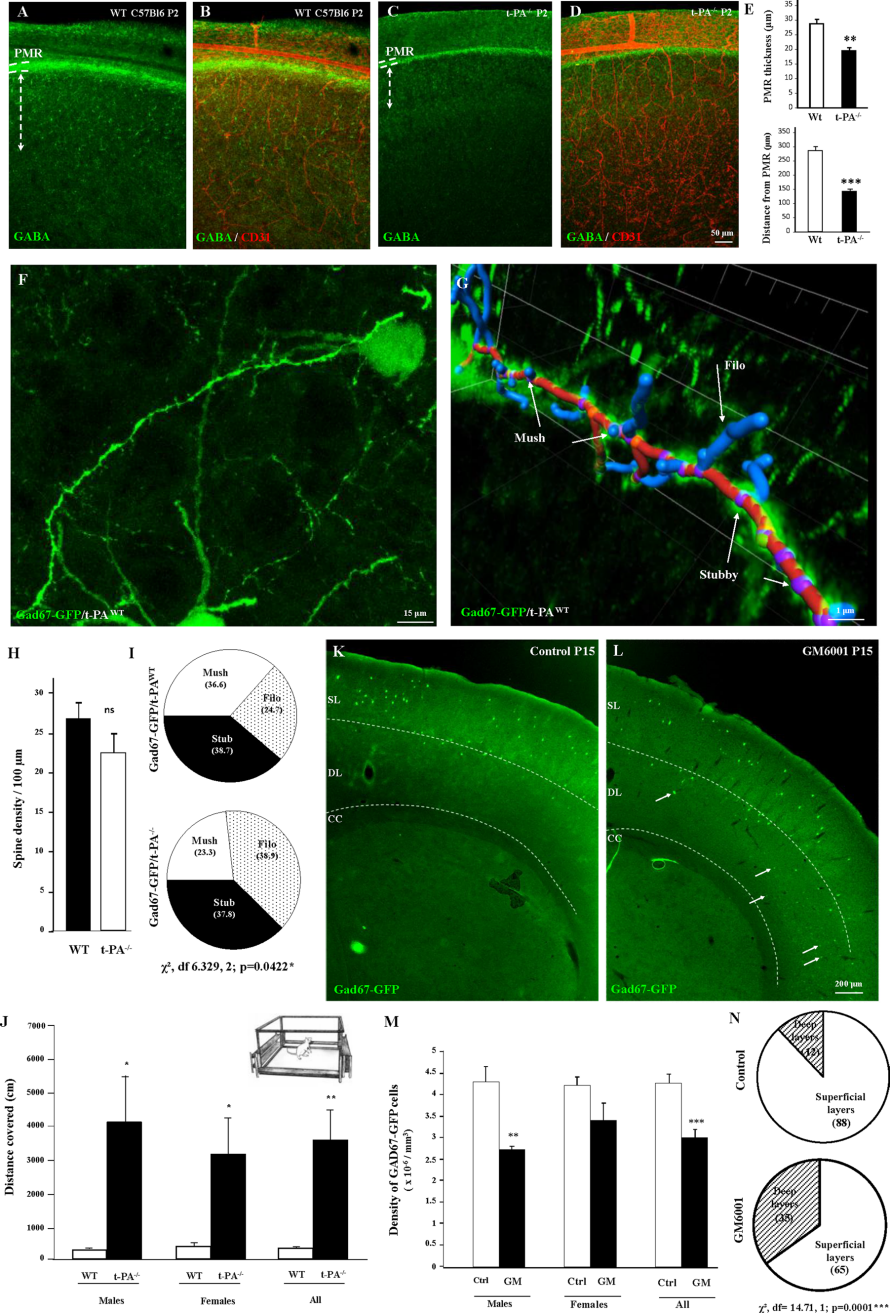
Effects of t-PA invalidation and in vivo GM6001 exposure on the cortical positioning and the spine morphology of GABA interneurons populating the superficial cortical layers. **a**–**e** Visualization of GABA interneurons (**a**–**c**) and microvessels (**b**, **d**) in P2 cortical slices from wild-type (**a**, **b**) and t-PA null (**c**, **d**) mice. Note the reduction of the PMR thickness (**b**, **d**, **e**, upper panel) and the reduced distance of GABA interneurons from the PMR (**b**, **d**, **e**, lower panel) in t-PA null mice. **f** Visualization of a Gad67-GFP neuron presenting dendritic spines in the superficial cortical layers of a wild-type mouse at P15. **g** Illustration of a graphic design obtained after spine analysis performed with the *Filament tracer* tool from the Imaris software. Three spine subtypes were analyzed: filopodia (Filo), mushrooms (Mush), and stubbies (Stub). Fiber is colored in red, mushrooms, and filopodia in blue, stubby in purple. **h**, **i** Quantification of the spine density (**h**) and the distribution of spine subtypes (**i**) in wild-type and t-PA^−/−^ mice at P15. **j** Quantification of the locomotor activity of wild-type and t-PA^−/−^ mice at P15. **k**–**m** Visualization (**k**, **l**) and quantification (**m**) of the positioning of Gad67-GFP neurons at P15 in control (**k**, **m**) and in vivo-exposed mice to the MMP inhibitor GM6001 (10 mg/kg; **l**, **m**). Note the presence of numerous ectopic cells in deep layers (**l**; arrows). **n** Quantification of the distribution of Gad67-GFP neurons between superficial and deep cortical layers in control and GM6001-treated mice. *ns* not significant; *p < 0.05; **p < 0.01; ***p < 0.001 vs wild-type or control mice. The tests used for the statistical analysis, the number of independent experiments, the number of measures per experiment, and *p* values are detailed in Table 1

To research developmental consequences of the impairment of t-PA and MMP activities characterized in pial and radial microvessels during perinatal life, we performed histological and behavioral studies in t-PA^−/−^ and double transgenic Gad67-GFP/t-PA^−/−^ mice (Fig. 6). Double immunolabeling experiments targeting GABA and CD31 revealed a marked reduction of the PMR thickness in P2 t-PA^−/−^ mice (*p* < 0.01; Fig. 6a–e). In addition, immunohistochemistry showed that, in t-PA^−/−^ mice, the distance of GABA interneurons from the PMR was significantly reduced (*p* < 0.001; Fig. 6a–e). Because a major phase of elimination/maturation of the GABAergic neurons has been described during the first 2 weeks postnatally [39], we investigated spine morphology of Gad67-GFP neurons at P15. Confocal microscopy acquisition in superficial layers II–IV of P15 Gad67-GFP/t-PA^wt^ revealed several GABAergic neurons with dendritic spines (Fig. 6f). The 3D image analysis with the Imaris *Filament tracer* application allowed discrimination of three subtypes of spines (mushrooms, filopodia, and stubbies; Fig. 6g). Quantification showed no significant differences in spine density between Gad67-GFP/t-PA^WT^ and Gad67-GFP/t-PA^−/−^ mice (Fig. 6h). In contrast, the distribution of spine subtypes was significantly impaired (Fig. 6i). In particular, the proportion of filopodia was increased in P15 Gad67-GFP/t-PA^−/−^ mice (*p* < 0.05; Fig. 6i). This effect on Gad67-GFP neurons was associated with a decrease of somatostatinergic neurons in the superficial layers of t-PA^−/−^ mice at P15 (Supplementary Fig. 7). Furthermore, behavioral experiments showed that morphometric and cellular impairments observed in t-PA^−/−^ mice at P15 were associated with locomotor troubles; indeed, a marked increase in the total distance covered was found in male and female t-PA^−/−^ mice at P15 (*p* < 0.01 for females and males; Fig. 6j). Previous reports indicated that in vivo administration of the MMP inhibitor GM6001 was able to impact vascular remodeling in mouse brain [40], and we consequently investigated the effects of a daily injection of GM6001 (10 mg/kg) during perinatal life in Gad67-GFP mice (Fig. 6k–n). As observed in t-PA^−/−^ mice, data revealed a marked reduction in the density of Gad67-GFP neurons at P15 in the superficial cortical layers of animals in vivo-exposed to GM6001 during perinatal life (*p* < 0.001 for females and males; Fig. 6k–m). In addition, several ectopic cells were observed in deep cortical layers (Fig. 6k, l, n; arrows). Altogether, these data indicate that impairment of MMP-9 and t-PA affects the positioning of GABAergic interneurons populating the superficial cortical layers and this cellular phenotype is associated with exacerbated locomotor activity in t-PA null mice.

### The positioning of GABA interneurons populating the superficial cortical layers is impaired in Grin1^*lox/lox*^/VeCad^*Cre*^ mice

**Fig. 7:**
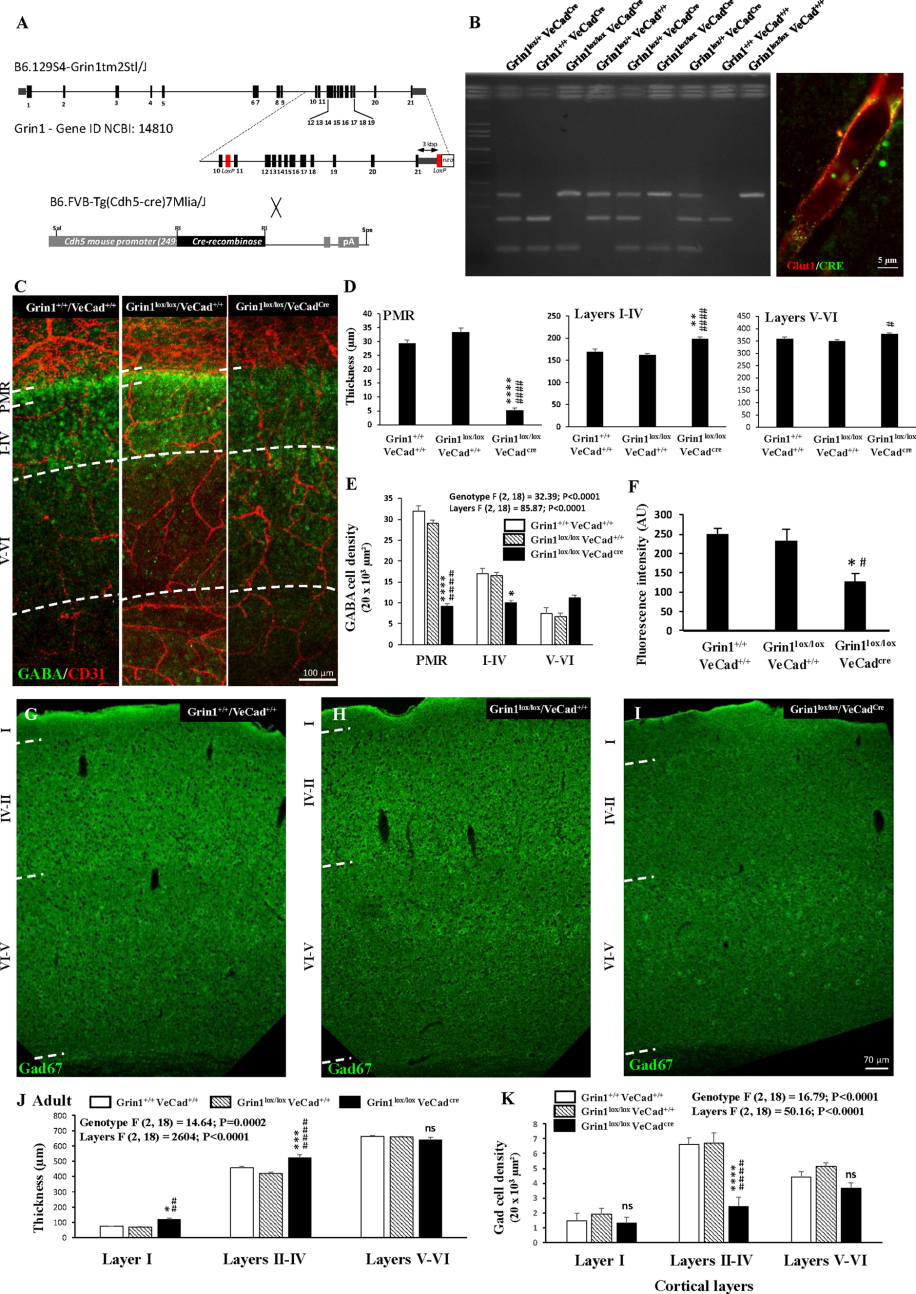
Effects of endothelial GluN1 invalidation on the pial migratory route and on the positioning of GABA interneurons. **a**, **b** Gene identity maps of floxed Grin1 and VE-Cadherin-Cre transgenic mice (**a**) used to generate Grin1^*lox/lox*^/VeCad^*Cre*^ transgenic mice (**b**; left panel). The endothelial expression of the Cre recombinase was controlled by immunohistochemistry (**b**; right panel). **c** Double immunohistochemistry experiments visualizing microvessels and GABA interneurons in the developing cortex of wild-type (Grin1^+*/*+^/VeCad^+*/*+^; left panel), Grin1^*lox/lox*^/VeCad^+*/*+^ (middle panel), and Grin1^*lox/lox*^/VeCad^*Cre*^ (right panel) mice at birth (P0). **d**, **e** Quantification of the thickness of the PMR, the developing superficial cortical layers (I–IV; **d**) and of the GABA interneuron density (**e**) in wild-type (Grin1^+*/*+^/VeCad^+*/*+^), Grin1^*lox/lox*^/VeCad^+*/*+^, and Grin1^*lox/lox*^/VeCad^*Cre*^ mice at birth. **f** Quantification by videomicroscopy of the effect of glutamate (100 µM) on the MMP-9-like activity in cortical slices from wild-type (Grin1^+*/*+^/VeCad^+*/*+^), Grin1^*lox/lox*^/VeCad^+*/*+^, and Grin1^*lox/lox*^/VeCad^*Cre*^ mice. Images of the in situ zymographies are provided in Supplementary Fig. 8. **g**–**i** Visualization at low magnification of the positioning of Gad67-positive neurons in the neocortex of adult wild-type (Grin1^+*/*+^/VeCad^+*/*+^; **g**), Grin1^*lox/lox*^/VeCad^+*/*+^ (**h**) and Grin1^*lox/lox*^/VeCad^*Cre*^ (**i**) mice. Note a depopulation of Gad67-immunoreactive cells in the superficial cortical layers in Grin1^*lox/lox*^/VeCad^*Cre*^ mice. **j** Quantification of the thickness of the layer I, superficial layers II–IV and deep layers V–VI in adult wild-type (Grin1^+*/*+^/VeCad^+*/*+^), Grin1^*lox/lox*^/VeCad^+*/*+^, and Grin1^*lox/lox*^/VeCad^*Cre*^ mice. **k** Quantification of the density of Gad-immunoreactive cells in the layer I, superficial layers II–IV and deep layers V–VI in adult wild-type (Grin1^+*/*+^/VeCad^+*/*+^), Grin1^*lox/lox*^/VeCad^+*/*+^, and Grin1^*lox/lox*^/VeCad^*Cre*^ mice. *ns* not significant; *p < 0.05; **p < 0.01; ***p < 0.001; ****p < 0.0001 vs wild-type mice. ^#^*p* < 0.05; ^##^*p* < 0.01; ^####^*p* < 0.0001 vs Grin1^*lox/lox*^/VeCad^+*/*+^. The tests used for the statistical analysis, the number of independent experiments, the number of measures per experiment, and *p* values are detailed in Table 1

Previous studies reported the expression of NMDA receptors by migrating GABAergic precursors [41]. To reinforce the demonstration of a direct contribution of the endothelial NMDA receptor on migrating GABA interneurons in the PMR and superficial cortical layers, we used Grin1^*lox/lox*^/VeCad^*Cre*^ mice. LoxP and Cre recombinase insertions were validated by PCR (Fig. 7a, b), while Grin1 inactivation was validated by qRT-PCR from purified cortical microvessel preparations (Supplementary Fig. 9). Major phenotypic abnormalities were observed regarding the positioning of GABA interneurons at two developmental stages. At birth, the thickness of the PMR was strongly reduced in Grin1^*lox/lox*^/VeCad^*Cre*^ mice when compared to Grin1^+*/*+^/VeCad^+*/*+^ (wild-type) and Grin1^*lox/lox*^/VeCad^+*/*+^ mice (*p* < 0.0001; Fig. 7c, d). This effect was associated with a significant decrease of the GABA interneuron density in the developing superficial layers (*p* < 0.0001; Fig. 7c, e). Moreover, in situ zymography experiments showed a significant decrease of the glutamate-induced MMP-like activity along the PMR (*p* < 0.05; Fig. 7f and Supplementary Fig. 8). Interestingly, an MMP-like activity was observed in nervous cells in Grin1^*lox/lox*^/VeCad^*Cre*^ mice (Supplementary Fig. 8; arrowheads). In adults, the cortical positioning of GABA interneurons remained clearly impaired in Grin1^*lox/lox*^/VeCad^*Cre*^ mice (Fig. 7h–i). In particular, a strong depopulation of Gad67-immunoreactive interneurons was observed and quantified in the superficial cortical layers II–IV (*p* < 0.0001; Fig. 7k). Interestingly, ectopic cells were observed in layer V and the thickness of layer I was significantly increased in Grin1^*lox/lox*^/VeCad^*Cre*^ mice (*p* < 0.0001; Fig. 7i, j). These results strongly support the contribution of the endothelial NMDA receptor for a correct positioning of GABA interneurons populating the superficial cortical layers.

## Discussion

**Fig. 8:**
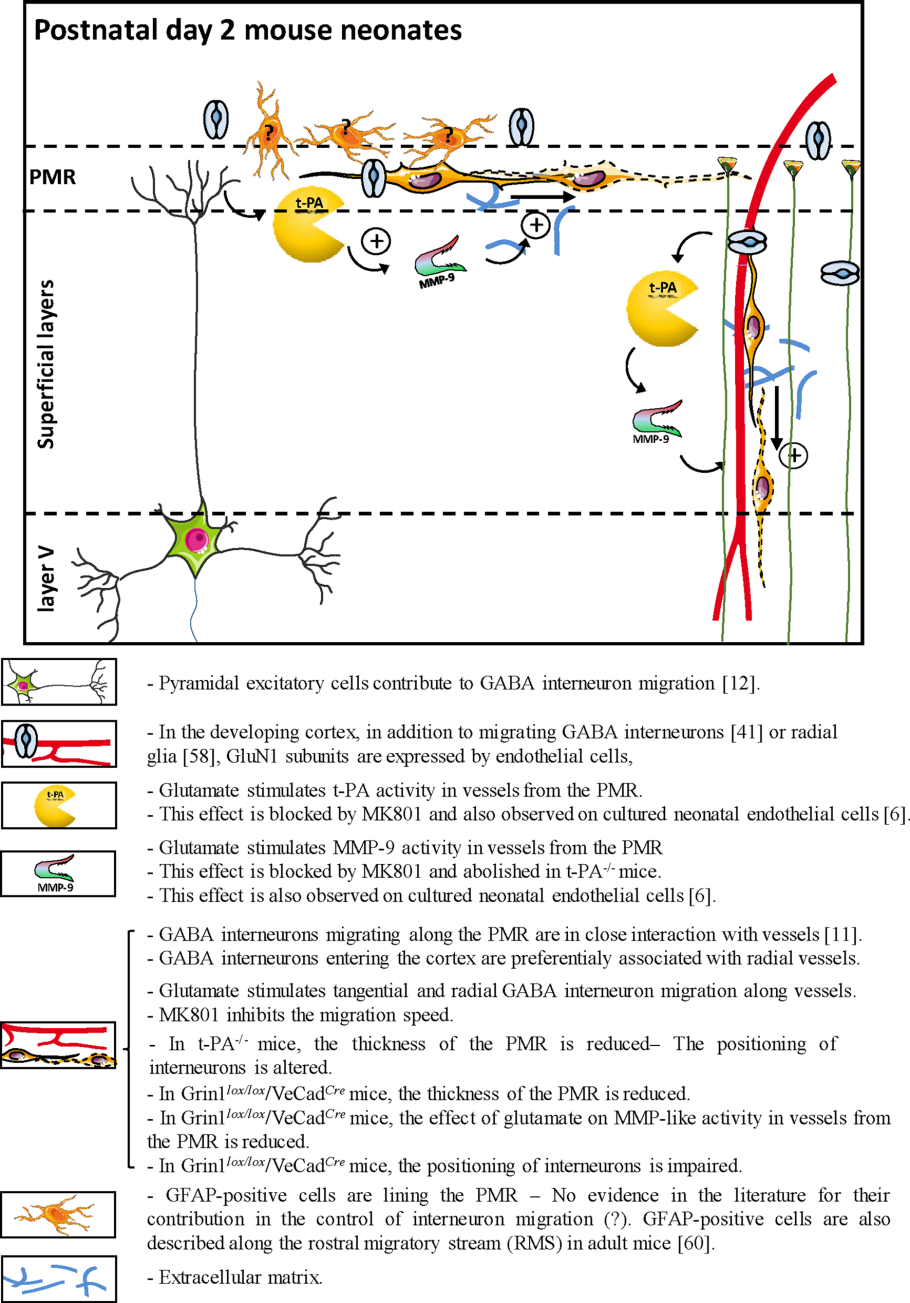
Graphical abstract integrating highlights from the present study and several studies from the literature positioning the contribution of the endothelial NMDA receptor in the regulation of MMP-9 activity and the vessel-associated migration of GABA interneuron arising from the PMR. Highlights are described and classified by graphical items. Data from the literature are mentioned by numbers in brackets which refer to the reference list of the main manuscript

Literature suggests that exposure of the developing brain to NMDA antagonists during perinatal life is deleterious for the survival of immature GABA interneurons [13, 16, 17]. In humans, as well as in rodents, numerous GABAergic interneurons are still migrating during late gestation and even after birth [9, 18]. This process has been shown to be vessel-associated (Fig. 8; [11]). Moreover, in vitro studies revealed age-specific expression of functional NMDA receptors by endothelial cells during perinatal life [6, 42] and, interestingly, at a pathological level, several recent studies reported links between NMDA receptor hypofunction during early life and GABA interneurons that could contribute to adult diseases [22–24]. Based on these statements, we hypothesized that glutamate would contribute to the control of the vasculo-associated migration of immature GABA interneurons by modulating the endothelial NMDA receptor.

Using WT and different transgenic (Gad67-GFP, Gad67-GFP/t-PA^−/−^, and Grin1^*lox/lox*^/VeCad^*Cre*^) mice, highlights of the present study (Fig. 8) are as follows: (1) glutamate induces an NMDA-dependent increase of MMP-9 and t-PA activities in microvessels along the pial migratory route and radial cortical microvessels in superficial layers, (2) there is a strong association of GABA interneurons with radial microvessels in the developing cortical layers, (3) glutamate induces an NMDA-dependent increase of the migration speed of immature GABA interneurons along the pial migratory route and radial cortical vessels, (4) effects of glutamate on MMP-9 activity is t-PA dependent and t-PA invalidation impairs the positioning of GABA interneurons in the superficial cortical layers, and (5) a marked reduction of the glutamate-induced MMP-9 activity is observed along the PMR in Grin1^*lox/lox*^/VeCad^*Cre*^ and this effect is associated with a depopulation of GABA interneurons in superficial cortical layers.

### Glutamate regulates MMP-9 and t-PA activities in cortical microvessels

Although the vasculo-associated migration of GABAergic interneurons along the pial migratory route has been clearly shown [10], the cellular interactions and molecular mechanisms involved remain poorly understood (Fig. 8). It has been shown that deletion of VEGF from endothelial cells resulted in impaired angiogenesis and marked alteration of the GABAergic cortical lamination reinforcing the contribution of microvessels to GABAergic neuronal migration [43]. Moreover, several studies showed that migration of GABAergic neurons requires a close relationship with excitatory pyramidal cells (Fig. 8; [12, 44]). For example, recent data revealed that neuregulin 3 expressed by pyramidal excitatory neurons acts as a chemoattractive factor for GABAergic interneurons [12]. Nevertheless, very few data are available regarding mechanisms able to regulate the vasculo-associated migration of GABA interneurons. Using transcriptomic, proteomic, and pharmacological approaches, we previously showed that endothelial cells from neonate mice present specific phenotypic characteristics and, in particular, express high levels of functional NMDA receptors [1, 6, 42]. In line with this notion, glutamate has been shown in pathological conditions such as stroke to exacerbate the release and recycling activity of the endothelial proteases MMP-9 [45] and t-PA [46, 47]. Taken together, these data suggest that excitatory neurons would be able to regulate endothelial cell activity not only in pathological conditions but also in a developmental context. Consistent with this hypothesis, the present study showed that both MMP-9 and t-PA immunoreactivities were detected in microvessels from the PMR, whereas gel and in situ zymography revealed that glutamate induced a dose-dependent and MK801-dependent increase of the MMP-9 and t-PA activities along vessels of the PMR. In addition, the effects of glutamate on the vessel MMP-9 activity were abrogated in t-PA^−/−^ mice. This regionalized pattern of the glutamate-induced proteolytic activity would reinforce the notion of a regional contribution of MMP9 on brain development and plasticity. Considering that previous studies from the literature showed that cultured cortical endothelial cells from mouse neonates express NMDA receptors and that glutamate stimulates the release and the activity of MMP-9 [6], the present data support that in mouse neonates, glutamate stimulates MMP-9 activity in vessels from the PMR through NMDA receptor-dependent and t-PA-dependent mechanisms.

### Glutamate stimulates the vessel-associated migration of GABA interneurons via an NMDA-dependent mechanism

In the mature brain, neurotransmitters are mainly released in synapses for communication between neurons [48]. However, during nervous system development, a trophic role of neurotransmitters implies neuronal release before their axons establish contacts with their target cells. Even if there is increasing evidence of such physiological roles, these extrasynaptic trophic roles are less understood [48]. Because MMP-9 is involved in proteolysis of the extracellular matrix [49], our data prompted us to investigate glutamate effects on the vasculo-dependent migration of GABA interneurons. Using immunohistochemistry performed from birth to P2, our data clearly showed a close interaction of GABA interneurons with microvessels of the cortical superficial layers. Video-tracking experiments indicated that glutamate was able to stimulate both tangential and radial vessel-associated migration, whereas MK801 induced an opposite effect. Consistent with these data, recent studies showed that neuronal projections from pyramidal cells are able to release factors such as neuregulin 3 that are involved in the control of GABA interneuron positioning [44, 50], and several lines of evidence indicated that glutamate released by excitatory cells in the developing brain can act as a guidance cue for migrating cells [51]. Altogether, the present data support that glutamate impacts endothelial protease activity via NMDA receptors and promotes the vessel-associated migration of GABA interneurons.

### t-PA invalidation and in vivo treatment with GM6001 alter the cortical positioning of GABA interneurons in superficial layers

During embryonic life, interneurons migrate tangentially and then switch their mode of migration from tangential to radial as they invade the cortical plate [10, 52]. Whereas cells from MGE and CGE take similar tangential migration paths with migrating above and below the cortical plate [52], an important sorting of GABA interneurons occurs during the first 2 postnatal weeks, leading to the final layer-specific positioning [39]. This developmental time-course prompts us to quantify the cortical positioning of GABA interneurons at P15. The results showed that in the superficial cortical layers, the density of Gad67-GFP interneurons as well as somatostatin-immunoreactive neurons was markedly reduced in both t-PA^−/−^ and GM6001-treated mice. In addition, because several studies reported dendritic spines in interneurons [53, 54], we analyzed spine morphology in the cortex of double-mutant Gad67-GFP/tPA^−/−^ mice. Data revealed that in t-PA null mice, the distribution of dendritic spines was significantly impaired with an increased proportion of filopodia. These morphometric impairments were associated with an exacerbated locomotor activity of t-PA^−/−^ mice at P15. Interestingly, several studies previously described delayed neuronal migration [37], modifications of the extracellular matrix [55], and long-term behavioral troubles of t-PA null mice related to disinhibition [56, 57]. All these data suggest that the dysregulation of MMPs and t-PA is associated with the impaired positioning of GABA interneurons populating the superficial cortical layers. However, even if our data showed strong t-PA and MMP-9 immunoreactivities/activities in vessels from the PMR, the fact that t-PA^−/−^ mice are not conditional animals open new avenues of research. Indeed, a neuronal expression of t-PA has been also described in the literature [58] and it would be tempting to investigate, for example by developing t-PA floxed mice, the contribution of neuronal vs endothelial t-PA in neurovascular development. In addition, these results also raise new questioning regarding whether the positioning of GABA interneurons would be impaired in animals perinatally exposed to factors with NMDA-antagonist properties such as ketamine.

### The positioning of GABA interneurons in the cortical superficial layers is impaired in Grin1 ^*lox/lox*^/VeCad^*Cre*^ mice

Previous in vitro studies revealed that neonatal endothelial cells express NMDA receptors [6]. In particular, glutamate was shown to induce a MK801-dependent activation of MMP-9 in cultured endothelial cells supporting a direct effect of glutamate on this cell type. However, because in the developing brain, NMDA receptors are expressed by numerous cell types including immature GABA interneurons [41], we characterized the positioning of GABA interneurons in Grin1^*lox/lox*^/VeCad^*Cre*^ mice. At birth, data revealed a marked reduction of the PMR thickness, a decrease of the MMP-9-like activity along vessels from the PMR and a significant depopulation of GABA interneurons in the developing superficial layers. Similarly, in adult Grin1^*lox/lox*^/VeCad^*Cre*^ mice, the positioning of GABA interneurons in the cortical superficial layers II–IV was markedly impaired with a strong decreased of Gad67 immunoreactive neurons. In addition, ectopic cells were found in the cortical layers V–VI. Interestingly, the presence of ectopic cells in deep layers was also observed in animals in vivo-exposed to GM6001. The presence of such ectopic cells in deep layers would be explained by the description in the literature of different (periventricular and pial) migratory routes of GABA interneurons and would suggest distinct mechanisms controlling cell migration [10]. Interestingly, in alcohol-exposed animal models, it has been also described a depopulation of GABA interneurons in superficial cortical layers, while ectopic cells were found in layer V [59]. Taken together, it is tempting to bring together our present data and the NMDA-antagonist properties of ethanol.

In conclusion, results from the present study support a role of the endothelial NMDA receptor in the control of the late vessel-associated migration of GABA interneurons populating the superficial cortical layers. At a neurodevelopmental level, they show that MMP-9 immunoreactivity is detected in microvessels along the PMR and that t-PA and the endothelial NMDA receptor actively contribute in the control of the MMP-9 activity as well as in the positioning of GABA interneurons in the superficial cortical layers. At a pathophysiological level, the present data reinforce the debate regarding the use of molecules with NMDA-antagonist properties such as anesthetics during perinatal life when a late vasculo-associated migration of interneurons still occurs.

### Electronic supplementary material

Below is the link to the electronic supplementary material.

**Supplementary Fig.** **1** Quantification of GluN1 mRNA expression in cortical microvessels isolated by laser capture microdissection. **A** Visualization at low magnification of microvessels from the pial, superficial and deep areas after labeling of P2 cortical slices with isolectin-TRITC. **B–E** Step by step illustrations of the protocol used for laser microdissection. Practically, microvessels were identified using the red fluorescence and a region of interest was delimited using the microdissection apparatus LMD7000, Leica Microsystems (green line; **B,C**). Afterwards, laser capture was initiated under transmitted light and the selected vessel was dropped in a plastic tube for small sample RNA extraction (**D,E**). **F** Typical qRT-PCR amplification curves for GAPDH, GluN1 and negative controls (NC) obtained from microdissected vessel cDNA. Statistical analysis revealed no significant differences of GluN1 expression between pial, superficial (subpial) and deep vessels (insert). The test used for the statistical analysis, the number of independent experiments, the number of measures per experiment and p values are detailed in Table 1 (TIFF 17730 kb)

**Supplementary Fig.** **2** Visualization and technical characteristics of a typical confocal acquisition showing vessel-associated GABA interneurons in the superficial cortical layers of the developing cortex at postnatal day 2. Interneurons and microvessels were immunolabeled using GABA and CD31 antibodies, respectively. **A** Visualization of a x/y plane from a z-stack acquisition. **B,C** Visualization of the corresponding y–z (**B**) and x–z (**C**) sections. Note the close interaction between the two cell types and the presence of GABA-immunoreactive processes lining vessels. **D** Confocal acquisition parameters corresponding to the images shown in **A-C**. **E** Visualization of the approach used to quantify the interneuron/vessel distances using the LAS AF Lite software from Leica. Distances were measured from the outer part of the neuron to the outer part of the vessel (TIFF 31485 kb)

**Supplementary Fig.** **3 A-C** Visualizing in the neocortex from adult mice of CD31 and alpha-smooth muscle actin (ACTA2) immunoreactivities at low (**A,B**) and high (**C**) magnifications. ACTA2-positive labeling is used to discriminate between arteries/arterioles and veins (ACTA2-negative) [61]. **D–E** Visualization of ACTA2 positive vessels in the developing cortex at postnatal day 2 (P2). While arteries are visible in pial vessels, no ACTA2 immunolabeling vessels are observed in the developing cortex suggesting that, at this developmental stage, the artery/vein phenotype of radial vessels is not yet established. **F** Large view showing the preferential association of GABA-immunoreactive neurons with radial vessels in the superficial cortical layers of the developing neocortex at P2 (TIFF 29751 kb)

**Supplementary Fig.** **4** Immunohistochemical characterization of the pial migratory route in mouse neonates. **A-C** Double immunolabeling experiments showing CD31-positive microvessels from the pial migratory route (arrow heads; **A**) and GFAP immunoreactive astrocytes (arrows; **B**). Overlay of both signals **(C)** indicates that astrocytes are lining the inner face of pial vessels just above the pial migratory route. Note the presence of small processes entering the neocortex. **D–F** Double immunolabeling experiments showing microvessels from the pial migratory route (arrowhead; **D**) and doublecortin-positive cells (arrows; **E**). Overlay (**F**) indicates that doublecortin immunoreactive cells are lining the inner face of the pial vessels. **G-I** Visualization at low magnification of GFAP-positive cells in the developing cortex at postnatal day 2 showing no clusterization along the PMR. **J-L** Confocal planes showing the relative positioning of DCX- (blue), GFAP- (yellow) and CD31- (green) immunoreactive cells along the PMR. In particular, the overlay **(L)** shows DCX-positive cells (blue, arrows) just below pial vessels (green) and GFAP-positive cells (yellow, arrowheads) (TIFF 29626 kb)

**Supplementary Fig.** **5 A** Visualization of the microvessel organization in the developing cortex of t-PA^−/−^ mice at P2. **B** Comparison of the orientation of cortical microvessels in wild-type (WT) and t-PA^−/−^ mice at P2. **C-E** In situ zymography using the quenched fluorogenic substrate DQ-gelatin-FITC (**D**) to visualize the effect of glutamate (100 µM) on the MMP gelatinase activity along the vessels from the PMR (arrows, **C, E**) on P2 cortical slices from wild-type mice. **F–H** In situ zymography visualizing the effect of glutamate (100 µM) on the MMP gelatinase activity (**G**) on P2 cortical slices from t-PA^−/−^ mice at P2. Note the weak fluorescence along the PMR (arrows; **F, H**). Quantification and statistical analysis are provided in Fig. 4I. The tests used for the statistical analysis, the number of independent experiments, the number of measures per experiment and p values are detailed in Table 1 (TIFF 20678 kb)

**Supplementary Fig.** **6** Positioning of Gad67-GFP interneurons in the mature cortex of transgenic mouse FVB-Tg(GadGFP)45704Swn. **A** Cresyl violet staining of the neocortex of adult Gad67-GFP mice. **B** Visualization of the positioning of Gad67-GFP interneurons. Note the preferential localization of the GFP expressing neurons in the superficial cortical layers (TIFF 12716 kb)

**Supplementary Fig.** **7** Effect of t-PA invalidation on the positioning of GABA interneurons populating the superficial cortical layers. **A-H** Microphotographs visualizing the eGFP (**A,D,F**), somatostatin (SST; **B**), calretinin (CR; **G**) and the overlays eGFP/SST (**C,E**) and eGFP/CR (**H**) in Gad67-GFP mice at P15. Arrows indicate co-labeled cells. Stars indicate SST^+^/GFP^−^ as well as CR^+^/GFP^−^ cells. Arrowheads indicate SST^−^/GFP^+^ as well as CR^−^/GFP^+^ cells. **I** Western blot quantification of somatostatin (SST) expression in the somatosensory cortex of wild-type and t-PA^−/−^ mice at P15. **J** Quantification of the density of primary neurites of SST interneurons in the superficial layers of the somatosensory cortex of wild-type and t-PA^−/−^ mice at P15. **K** Quantification of the density of SST interneurons in the superficial cortical layers in wild-type and t-PA^−/−^ mice at P15. *p < 0.05; **p < 0.01; ***p < 0.001 vs wt. The tests used for the statistical analysis, the number of independent experiments, the number of measures per experiment and p values are detailed in Table 1 (TIFF 27505 kb)

**Supplementary Fig.** **8 A-E** Visualization of the MMP-9-like activity in pial vessels along the PMR in cultured slices from Grin1^+*/*+^/VeCad^+*/*+^ (WT), Grin1^*lox/lox*^/VeCad^+/+^ and Grin1^*lox/lox*^ VeCad^*Cre*^ mouse neonates. Microvessels were visualized using isolectin-TRITC **(A,C,E)** and gelatinase activity was visualized by incubating the slices with the DQ-gelatin-FITC substrate in presence of glutamate (100 µM; **B,D,E**). Note that in Grin1^*lox/lox*^/VeCad^*Cre*^ mice and contrasting to Grin1^*lox/lox*^/VeCad^+/+^ mice, the MMP-9-like activity is markedly reduced (arrow; **D–F**). The quantification of in situ zymography experiments is provided in Fig. 7F of the main manuscript. Interestingly, in Grin1^*lox/lox*^/VeCad^*Cre*^ mice a non-vascular cell activity was observed (arrowheads) in deepest layers (TIFF 8645 kb)

**Supplementary Fig.** **9** Validation of the *Grin1* primers designed for qRT-PCR experiments on brain microvessel extracts from Grin1^lox/lox^/VeCad^+/+^ and Grin1^lox/lox^/VeCad^Cre^ mice. **A** Visualization of the positioning of several pairs of primers at the exon junction 18-19. The primer pair shown in red was used for qRT-PCR experiments. **B** Sequences and characteristics of the selected primer pair. **C** Amplification curves obtained from 1/10, 1/100 and 1/1000 dilutions of cDNA extracts. **D** Standard curve from the amplification plot. **E** Melt curve plot. **F** Quantification by qRT-PCR of *Grin1*, *Grin2a* and *Grin2b* expression in microvessel extracts from Grin1^*lox/lox*^/VeCad^+/+^ and Grin1^*lox/lox*^/VeCad^*Cre*^ mice. Error bars represent SEM of 3 determinations from microvessel cortical cDNA extracts prepared from a pool of 20 animals per group. Because for two of three determinations *Grin1* was not detected (> 40 ct), we didn’t calculate mean values for this subunit. Only the value from the third determination is shown (TIFF 23603 kb)

**Supplementary Fig.** **10** Visualization at high magnification of Gad67-positive neurons in the layers IV and V of the neocortex from Grin1^+/+^/VeCad^+/+^ (**A**) and Grin1^lox/lox^/VeCad^Cre^ (**B**) mice. Note the depopulation of Gad67 immunoreactive cells in mutant mice. **C,D** Cresyl violet staining and NeuN immunoreactive cells visualizing the cortical layering in Grin1^+/+^/VeCad^+/+^ (**C**) and Grin1^lox/lox^/VeCad^Cre^ (**D**) in adult mice. Note the increased thickness of layer I and the decrease of Cresyl violet-stained cell bodies in Grin1^lox/lox^/VeCad^Cre^ mice. No layering differences were observed between inter- and intra-littermate control mice (data not shown) (TIFF 25455 kb)

Supplementary material 11 (DOCX 18 kb)

Supplementary material 12 (DOCX 28 kb)

Supplementary material 13 (AVI 19344 kb)

## Abbreviations

aCSF: Artificial cerebrospinal fluid
CR: Calretinin
DCX: Doublecortin
MMP-9: Matrix metalloproteinase 9
PAI-1: Plasminogen activator inhibitor 1
PMR: Pial migratory route
SST: Somatostatin
t-PA: Tissue-plasminogen activator
