# Supplementary figures and images for: Glutamate controls vessel-associated migration of GABA interneurons from the pial migratory route via NMDA receptors and endothelial protease activation
Source: Cell Mol Life Sci. 2019 Aug 7;77(10):1959–86. doi: 10.1007/s00018-019-03248-5 (PMC7229000; doi:10.1007/s00018-019-03248-5)

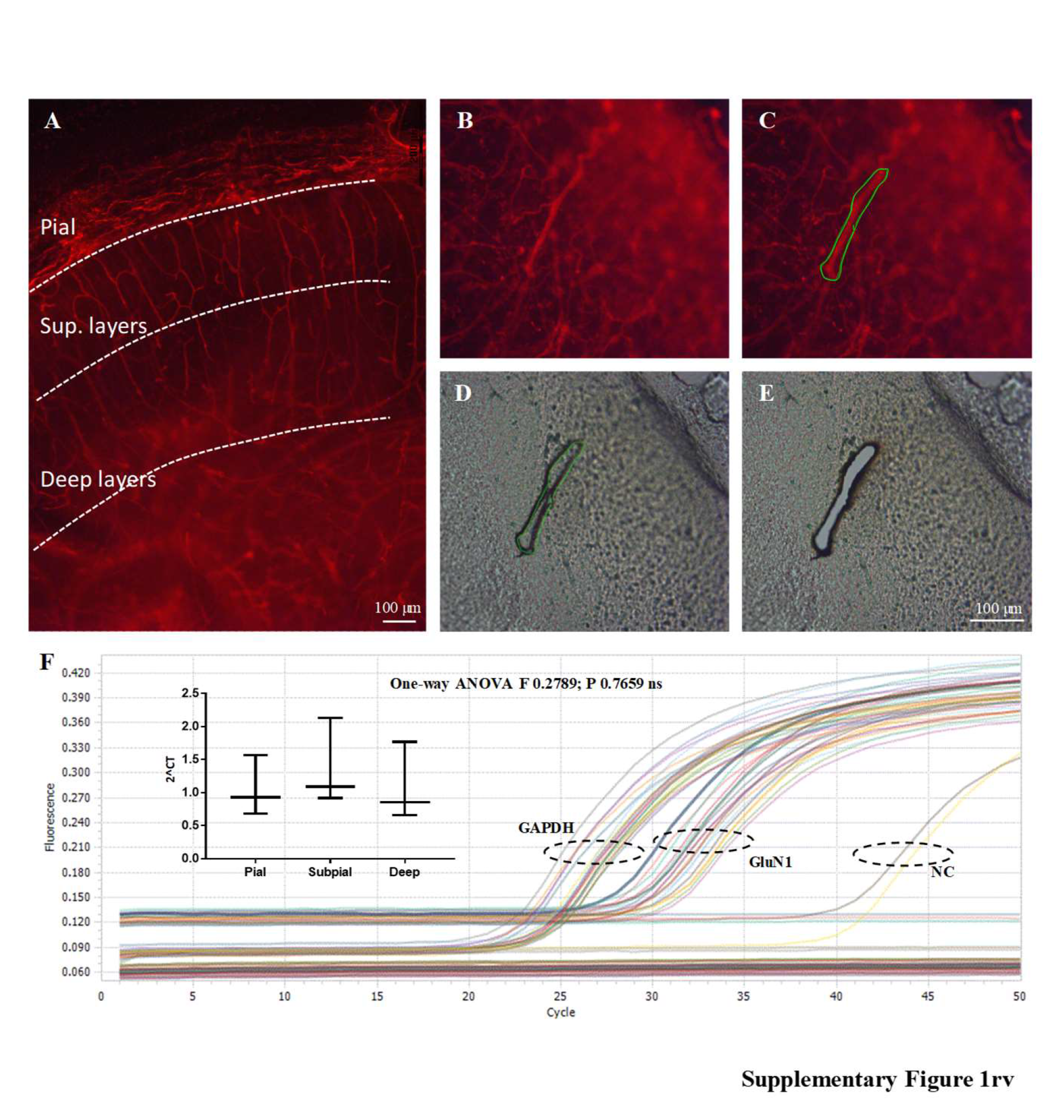

Supplement: Supplementary file 1 — Supplementary Fig. 1 Quantification of GluN1 mRNA expression in cortical microvessels isolated by laser capture microdissection. A Visualization at low magnification of microvessels from the pial, superficial and deep areas after labeling of P2 cortical slices with isolectin-TRITC. B–E Step by step illustrations of the protocol used for laser microdissection. Practically, microvessels were identified using the red fluorescence and a region of interest was delimited using the microdissection apparatus LMD7000, Leica Microsystems (green line; B,C). Afterwards, laser capture was initiated under transmitted light and the selected vessel was dropped in a plastic tube for small sample RNA extraction (D,E). F Typical qRT-PCR amplification curves for GAPDH, GluN1 and negative controls (NC) obtained from microdissected vessel cDNA. Statistical analysis revealed no significant differences of GluN1 expression between pial, superficial (subpial) and deep vessels (insert). The test used for the statistical analysis, the number of independent experiments, the number of measures per experiment and p values are detailed in Table 1 (TIFF 17730 kb) [file 18_2019_3248_MOESM1_ESM.tif]

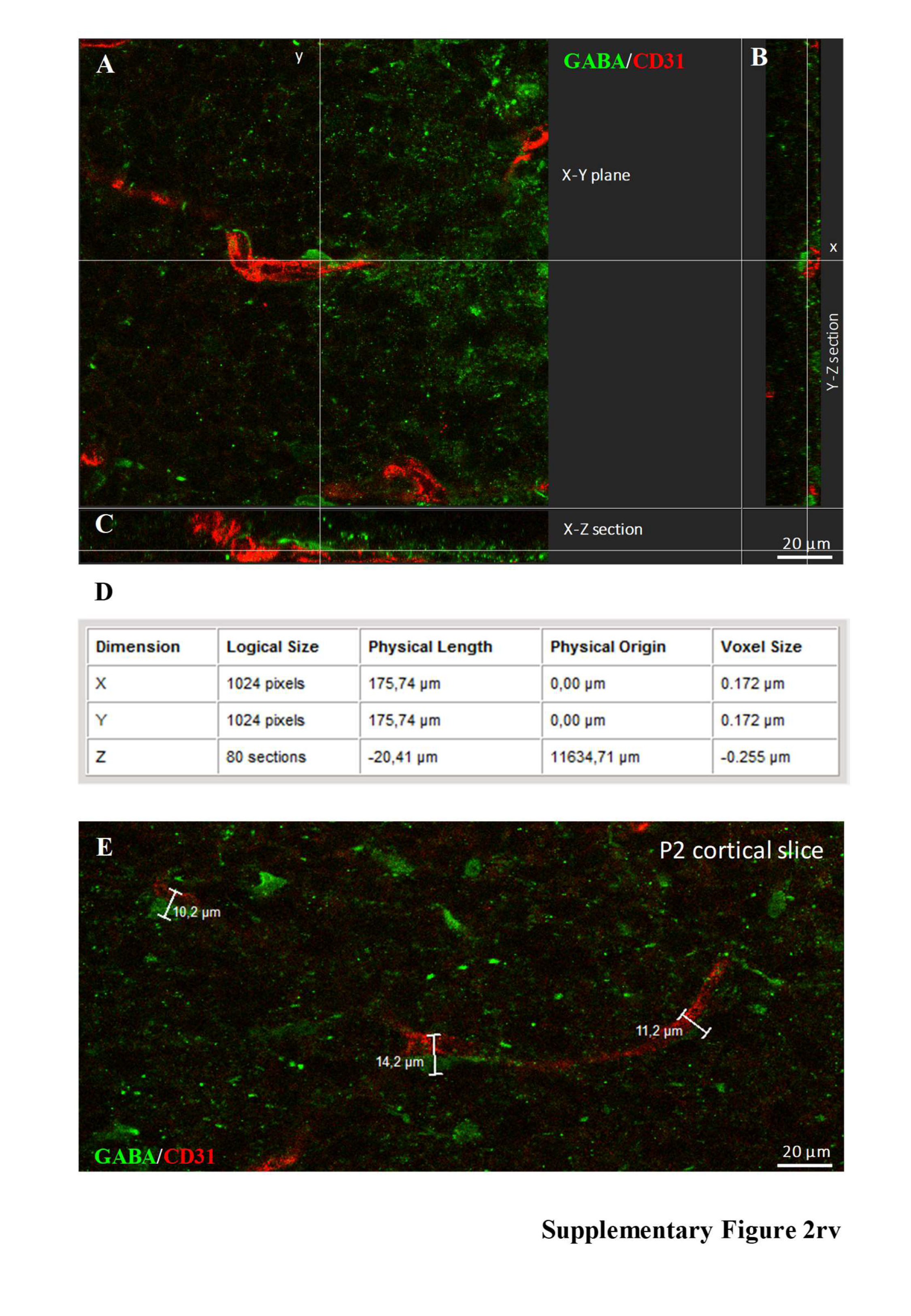

Supplement: Supplementary file 2 — Supplementary Fig. 2 Visualization and technical characteristics of a typical confocal acquisition showing vessel-associated GABA interneurons in the superficial cortical layers of the developing cortex at postnatal day 2. Interneurons and microvessels were immunolabeled using GABA and CD31 antibodies, respectively. A Visualization of a x/y plane from a z-stack acquisition. B,C Visualization of the corresponding y–z (B) and x–z (C) sections. Note the close interaction between the two cell types and the presence of GABA-immunoreactive processes lining vessels. D Confocal acquisition parameters corresponding to the images shown in A-C. E Visualization of the approach used to quantify the interneuron/vessel distances using the LAS AF Lite software from Leica. Distances were measured from the outer part of the neuron to the outer part of the vessel (TIFF 31485 kb) [file 18_2019_3248_MOESM2_ESM.tif]

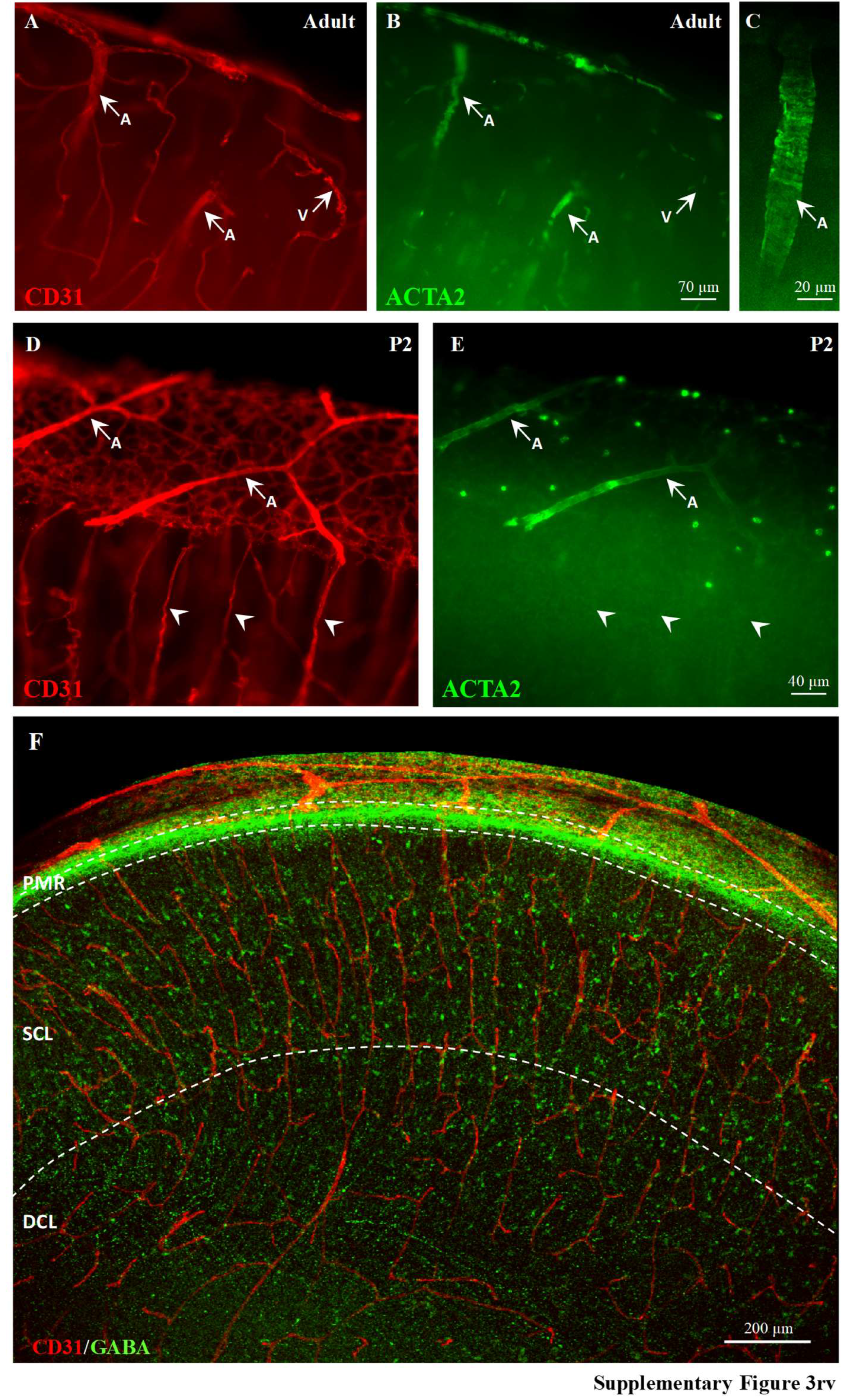

Supplement: Supplementary file 3 — Supplementary Fig. 3 A-C Visualizing in the neocortex from adult mice of CD31 and alpha-smooth muscle actin (ACTA2) immunoreactivities at low (A,B) and high (C) magnifications. ACTA2-positive labeling is used to discriminate between arteries/arterioles and veins (ACTA2-negative) [61]. D–E Visualization of ACTA2 positive vessels in the developing cortex at postnatal day 2 (P2). While arteries are visible in pial vessels, no ACTA2 immunolabeling vessels are observed in the developing cortex suggesting that, at this developmental stage, the artery/vein phenotype of radial vessels is not yet established. F Large view showing the preferential association of GABA-immunoreactive neurons with radial vessels in the superficial cortical layers of the developing neocortex at P2 (TIFF 29751 kb) [file 18_2019_3248_MOESM3_ESM.tif]

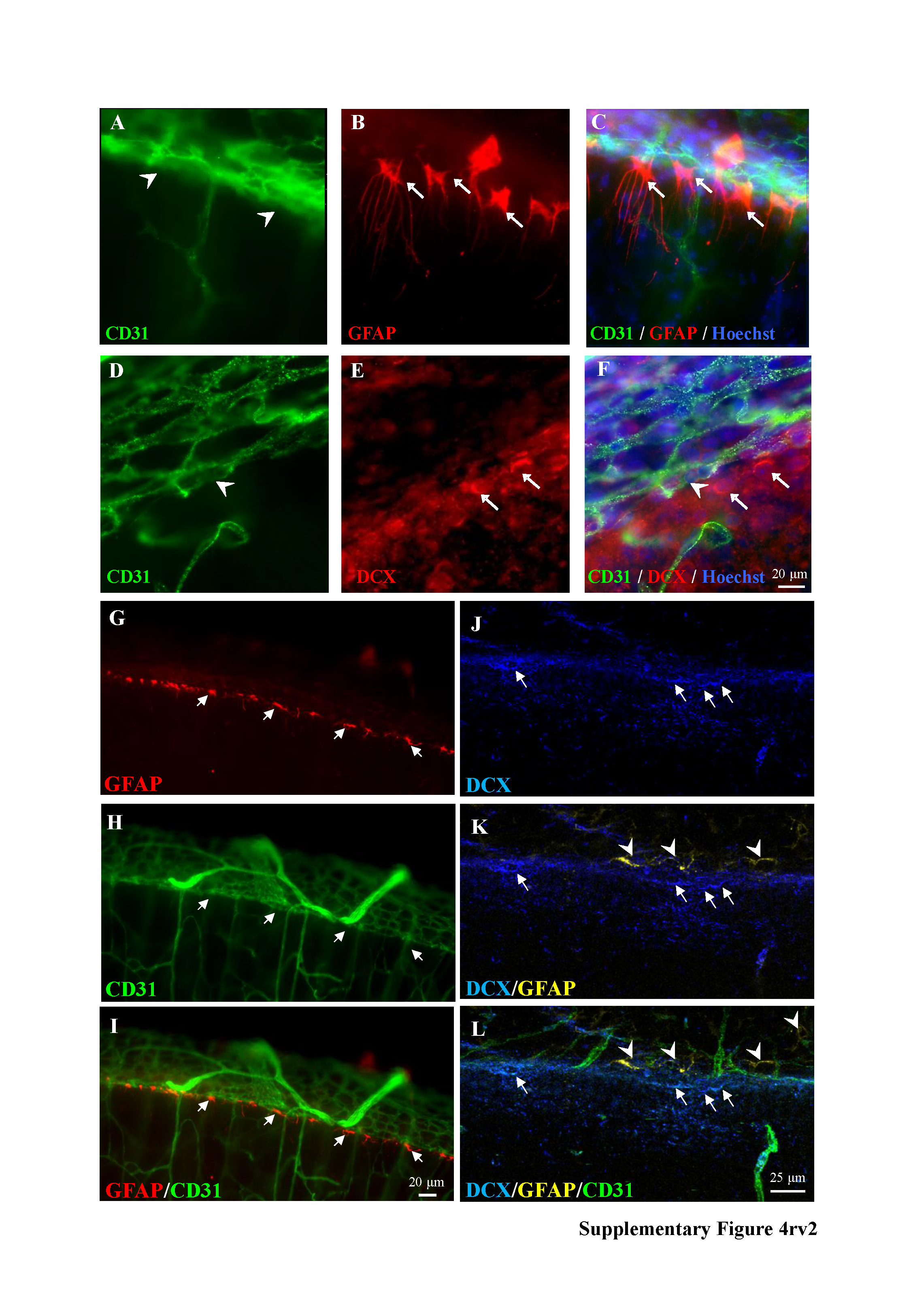

Supplement: Supplementary file 4 — Supplementary Fig. 4 Immunohistochemical characterization of the pial migratory route in mouse neonates. A-C Double immunolabeling experiments showing CD31-positive microvessels from the pial migratory route (arrow heads; A) and GFAP immunoreactive astrocytes (arrows; B). Overlay of both signals (C) indicates that astrocytes are lining the inner face of pial vessels just above the pial migratory route. Note the presence of small processes entering the neocortex. D–F Double immunolabeling experiments showing microvessels from the pial migratory route (arrowhead; D) and doublecortin-positive cells (arrows; E). Overlay (F) indicates that doublecortin immunoreactive cells are lining the inner face of the pial vessels. G-I Visualization at low magnification of GFAP-positive cells in the developing cortex at postnatal day 2 showing no clusterization along the PMR. J-L Confocal planes showing the relative positioning of DCX- (blue), GFAP- (yellow) and CD31- (green) immunoreactive cells along the PMR. In particular, the overlay (L) shows DCX-positive cells (blue, arrows) just below pial vessels (green) and GFAP-positive cells (yellow, arrowheads) (TIFF 29626 kb) [file 18_2019_3248_MOESM4_ESM.tif]

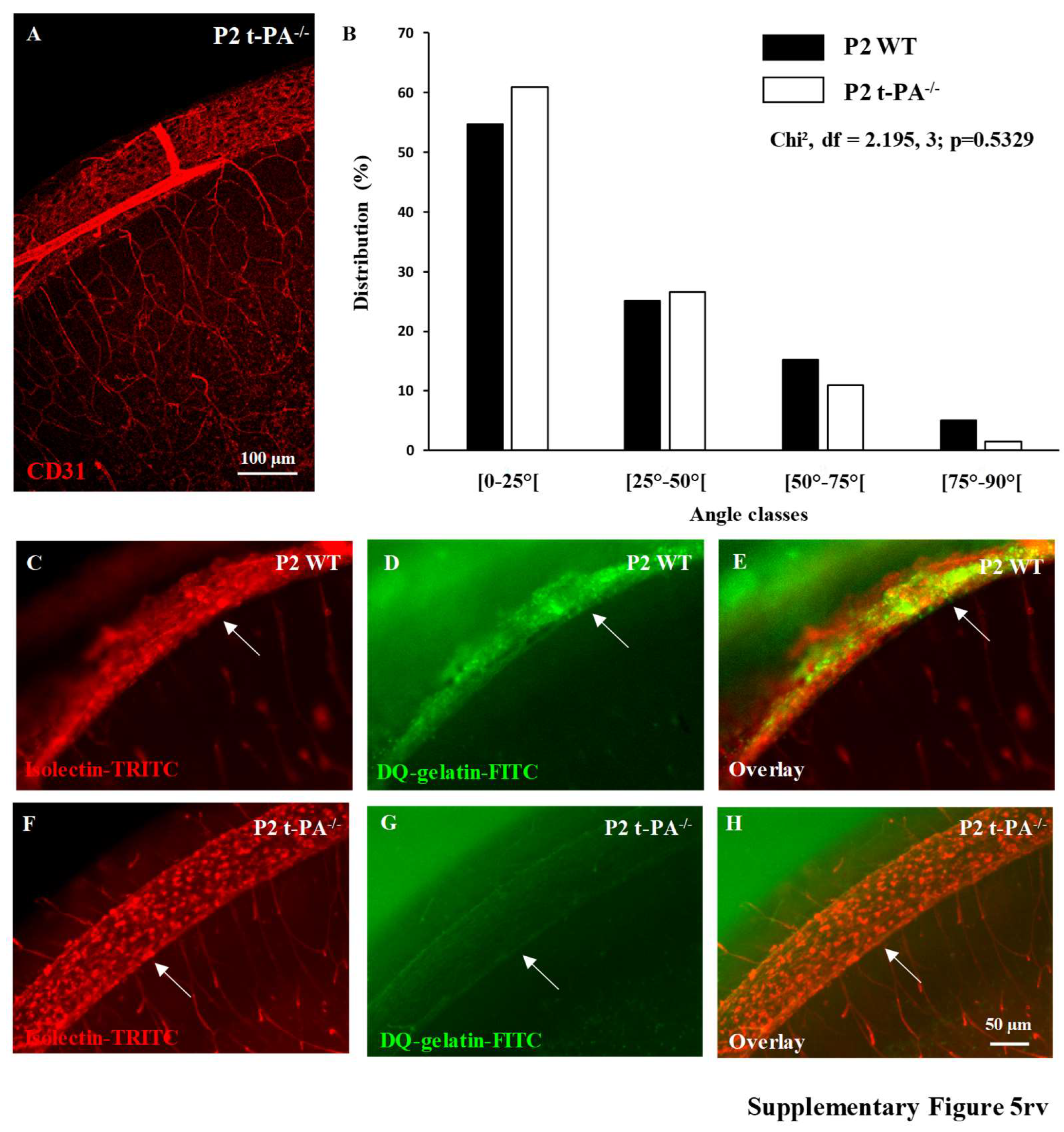

Supplement: Supplementary file 5 — Supplementary Fig. 5 A Visualization of the microvessel organization in the developing cortex of t-PA−/− mice at P2. B Comparison of the orientation of cortical microvessels in wild-type (WT) and t-PA−/− mice at P2. C-E In situ zymography using the quenched fluorogenic substrate DQ-gelatin-FITC (D) to visualize the effect of glutamate (100 µM) on the MMP gelatinase activity along the vessels from the PMR (arrows, C, E) on P2 cortical slices from wild-type mice. F–H In situ zymography visualizing the effect of glutamate (100 µM) on the MMP gelatinase activity (G) on P2 cortical slices from t-PA−/− mice at P2. Note the weak fluorescence along the PMR (arrows; F, H). Quantification and statistical analysis are provided in Fig. 4I. The tests used for the statistical analysis, the number of independent experiments, the number of measures per experiment and p values are detailed in Table 1 (TIFF 20678 kb) [file 18_2019_3248_MOESM5_ESM.tif]

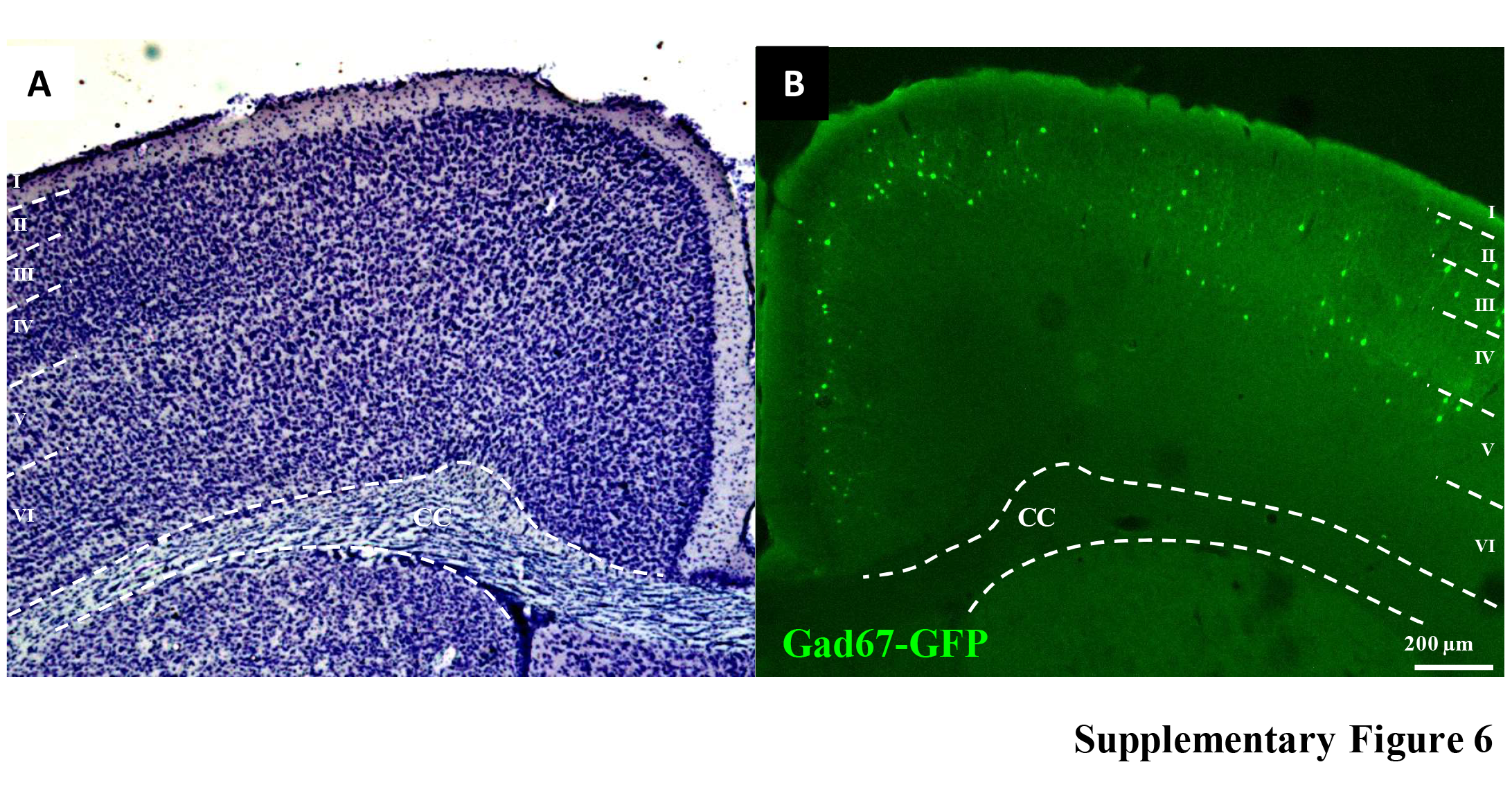

Supplement: Supplementary file 6 — Supplementary Fig. 6 Positioning of Gad67-GFP interneurons in the mature cortex of transgenic mouse FVB-Tg(GadGFP)45704Swn. A Cresyl violet staining of the neocortex of adult Gad67-GFP mice. B Visualization of the positioning of Gad67-GFP interneurons. Note the preferential localization of the GFP expressing neurons in the superficial cortical layers (TIFF 12716 kb) [file 18_2019_3248_MOESM6_ESM.tif]

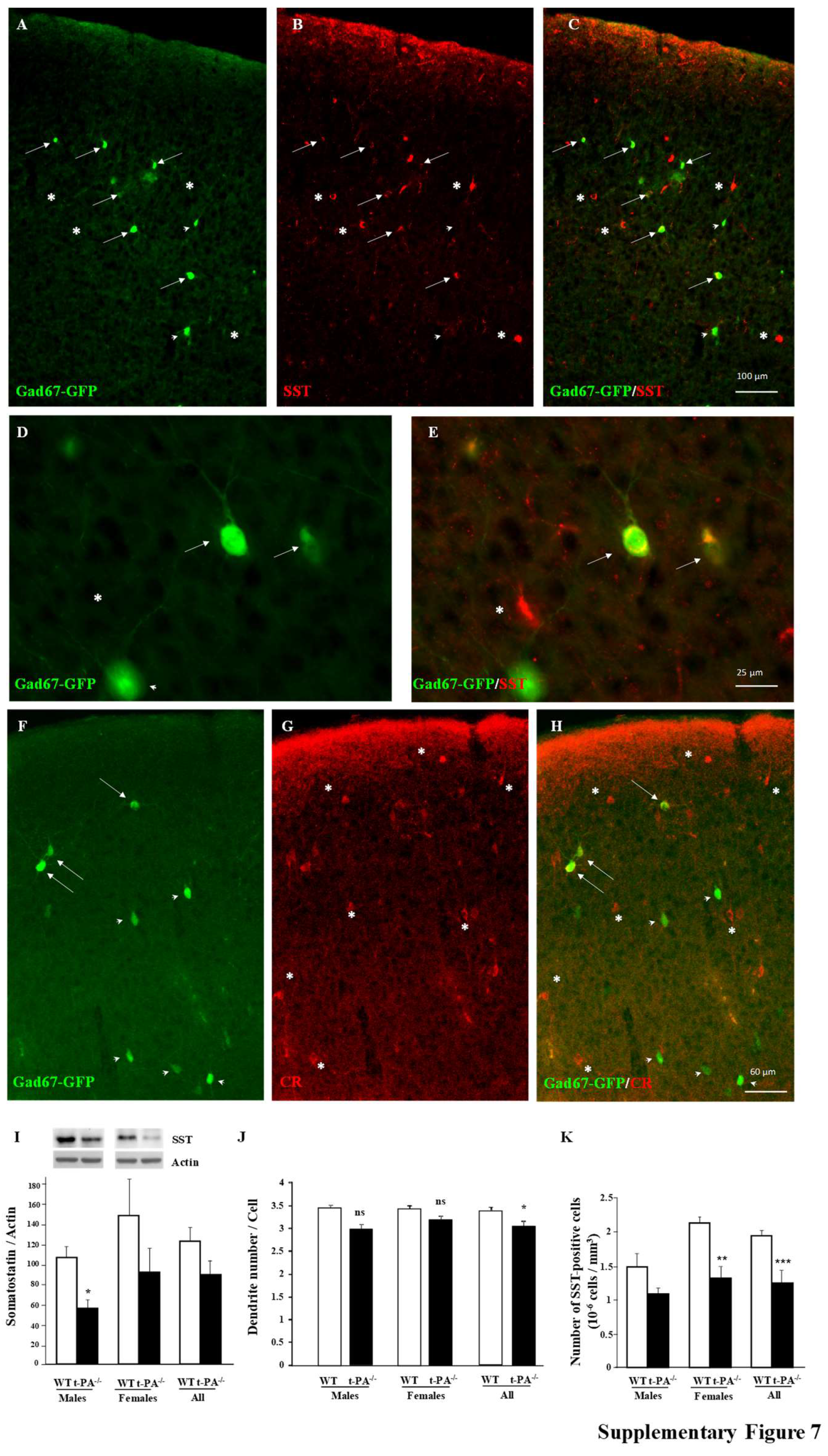

Supplement: Supplementary file 7 — Supplementary Fig. 7 Effect of t-PA invalidation on the positioning of GABA interneurons populating the superficial cortical layers. A-H Microphotographs visualizing the eGFP (A,D,F), somatostatin (SST; B), calretinin (CR; G) and the overlays eGFP/SST (C,E) and eGFP/CR (H) in Gad67-GFP mice at P15. Arrows indicate co-labeled cells. Stars indicate SST+/GFP− as well as CR+/GFP− cells. Arrowheads indicate SST−/GFP+ as well as CR−/GFP+ cells. I Western blot quantification of somatostatin (SST) expression in the somatosensory cortex of wild-type and t-PA−/− mice at P15. J Quantification of the density of primary neurites of SST interneurons in the superficial layers of the somatosensory cortex of wild-type and t-PA−/− mice at P15. K Quantification of the density of SST interneurons in the superficial cortical layers in wild-type and t-PA−/− mice at P15. *p < 0.05; **p < 0.01; ***p < 0.001 vs wt. The tests used for the statistical analysis, the number of independent experiments, the number of measures per experiment and p values are detailed in Table 1 (TIFF 27505 kb) [file 18_2019_3248_MOESM7_ESM.tif]

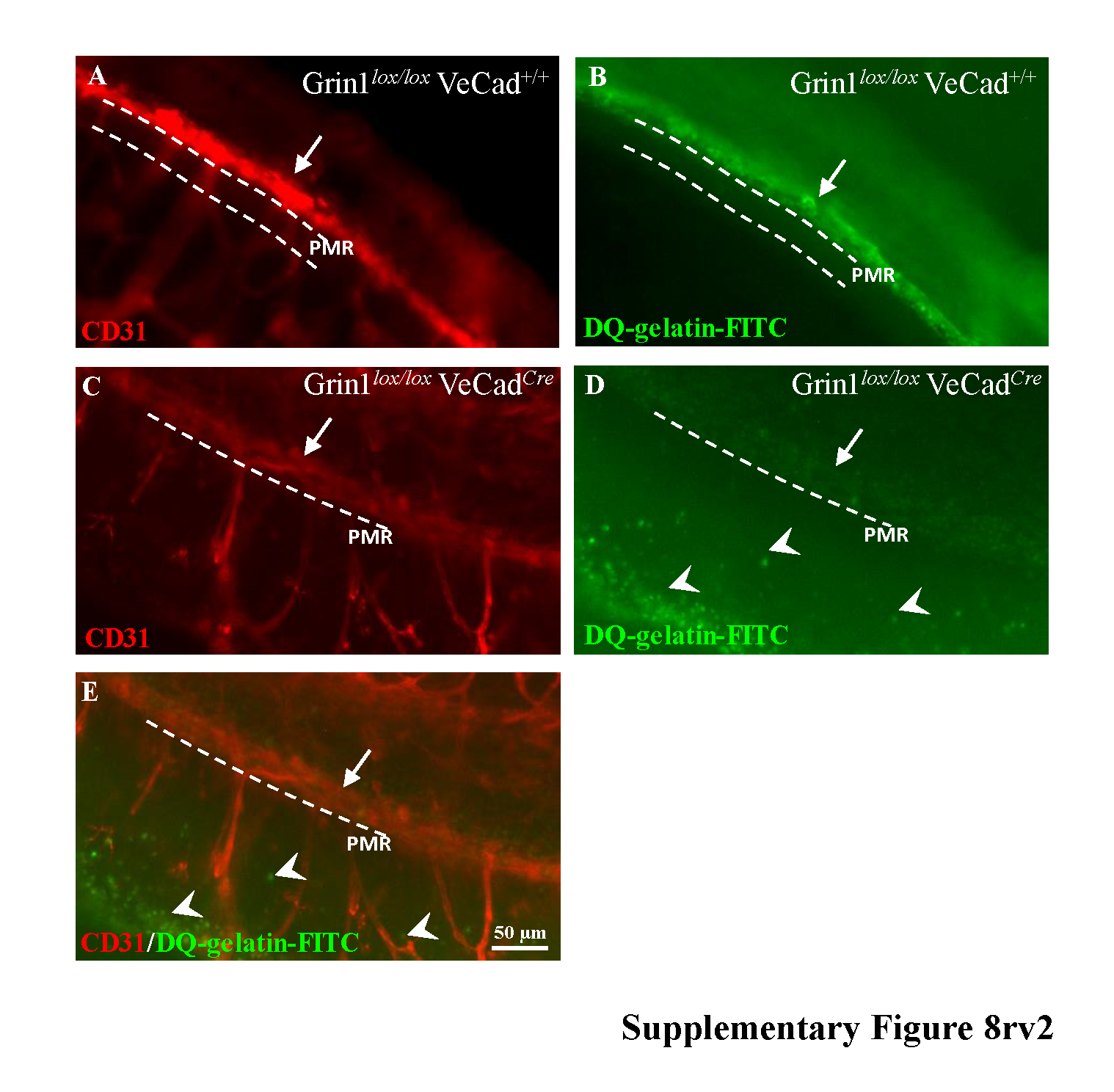

Supplement: Supplementary file 8 — Supplementary Fig. 8 A-E Visualization of the MMP-9-like activity in pial vessels along the PMR in cultured slices from Grin1+/+/VeCad+/+ (WT), Grin1lox/lox/VeCad+/+ and Grin1lox/lox VeCadCre mouse neonates. Microvessels were visualized using isolectin-TRITC (A,C,E) and gelatinase activity was visualized by incubating the slices with the DQ-gelatin-FITC substrate in presence of glutamate (100 µM; B,D,E). Note that in Grin1lox/lox/VeCadCre mice and contrasting to Grin1lox/lox/VeCad+/+ mice, the MMP-9-like activity is markedly reduced (arrow; D–F). The quantification of in situ zymography experiments is provided in Fig. 7F of the main manuscript. Interestingly, in Grin1lox/lox/VeCadCre mice a non-vascular cell activity was observed (arrowheads) in deepest layers (TIFF 8645 kb) [file 18_2019_3248_MOESM8_ESM.tif]

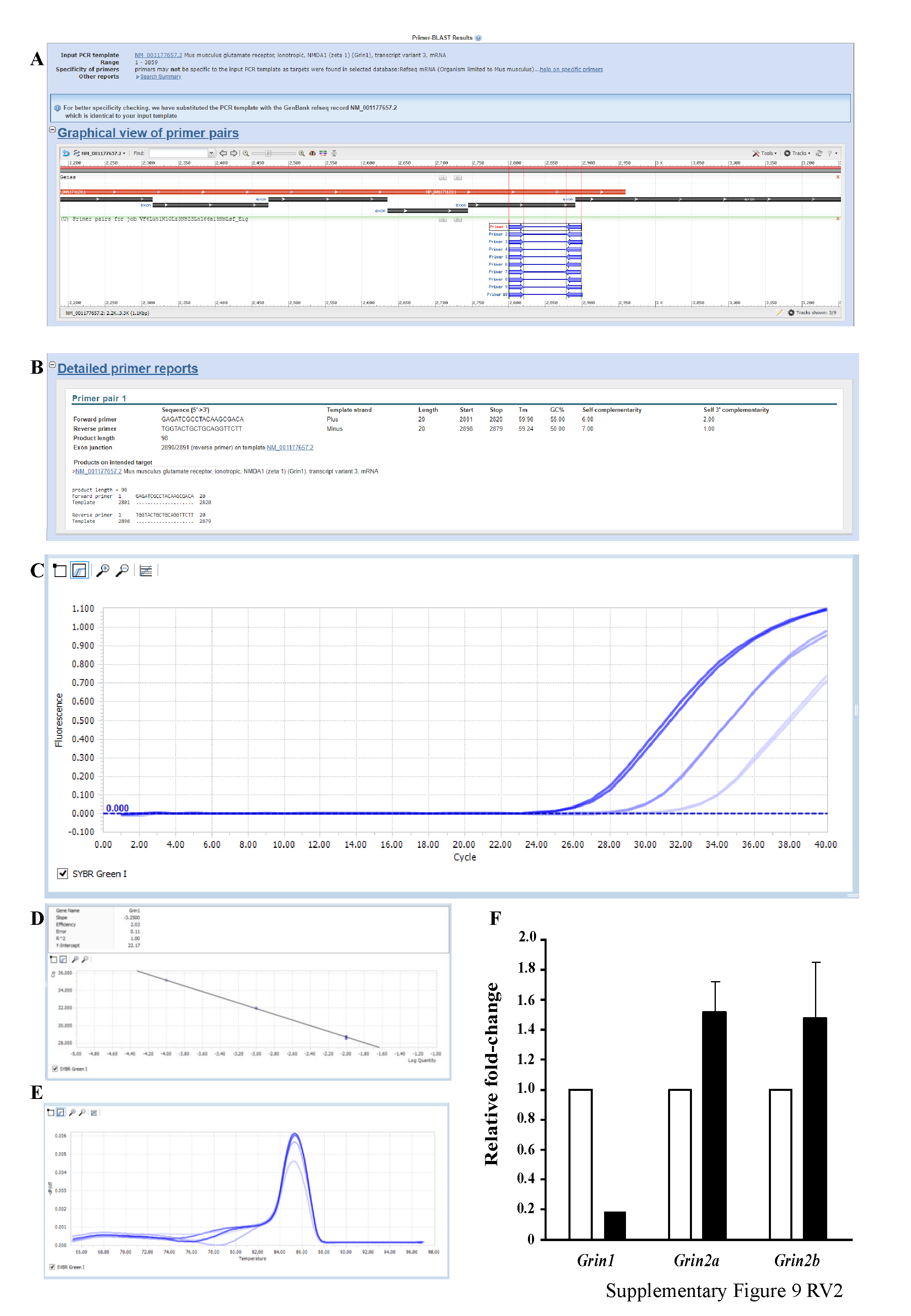

Supplement: Supplementary file 9 — Supplementary Fig. 9 Validation of the Grin1 primers designed for qRT-PCR experiments on brain microvessel extracts from Grin1lox/lox/VeCad+/+ and Grin1lox/lox/VeCadCre mice. A Visualization of the positioning of several pairs of primers at the exon junction 18-19. The primer pair shown in red was used for qRT-PCR experiments. B Sequences and characteristics of the selected primer pair. C Amplification curves obtained from 1/10, 1/100 and 1/1000 dilutions of cDNA extracts. D Standard curve from the amplification plot. E Melt curve plot. F Quantification by qRT-PCR of Grin1, Grin2a and Grin2b expression in microvessel extracts from Grin1lox/lox/VeCad+/+ and Grin1lox/lox/VeCadCre mice. Error bars represent SEM of 3 determinations from microvessel cortical cDNA extracts prepared from a pool of 20 animals per group. Because for two of three determinations Grin1 was not detected (> 40 ct), we didn’t calculate mean values for this subunit. Only the value from the third determination is shown (TIFF 23603 kb) [file 18_2019_3248_MOESM9_ESM.tif]

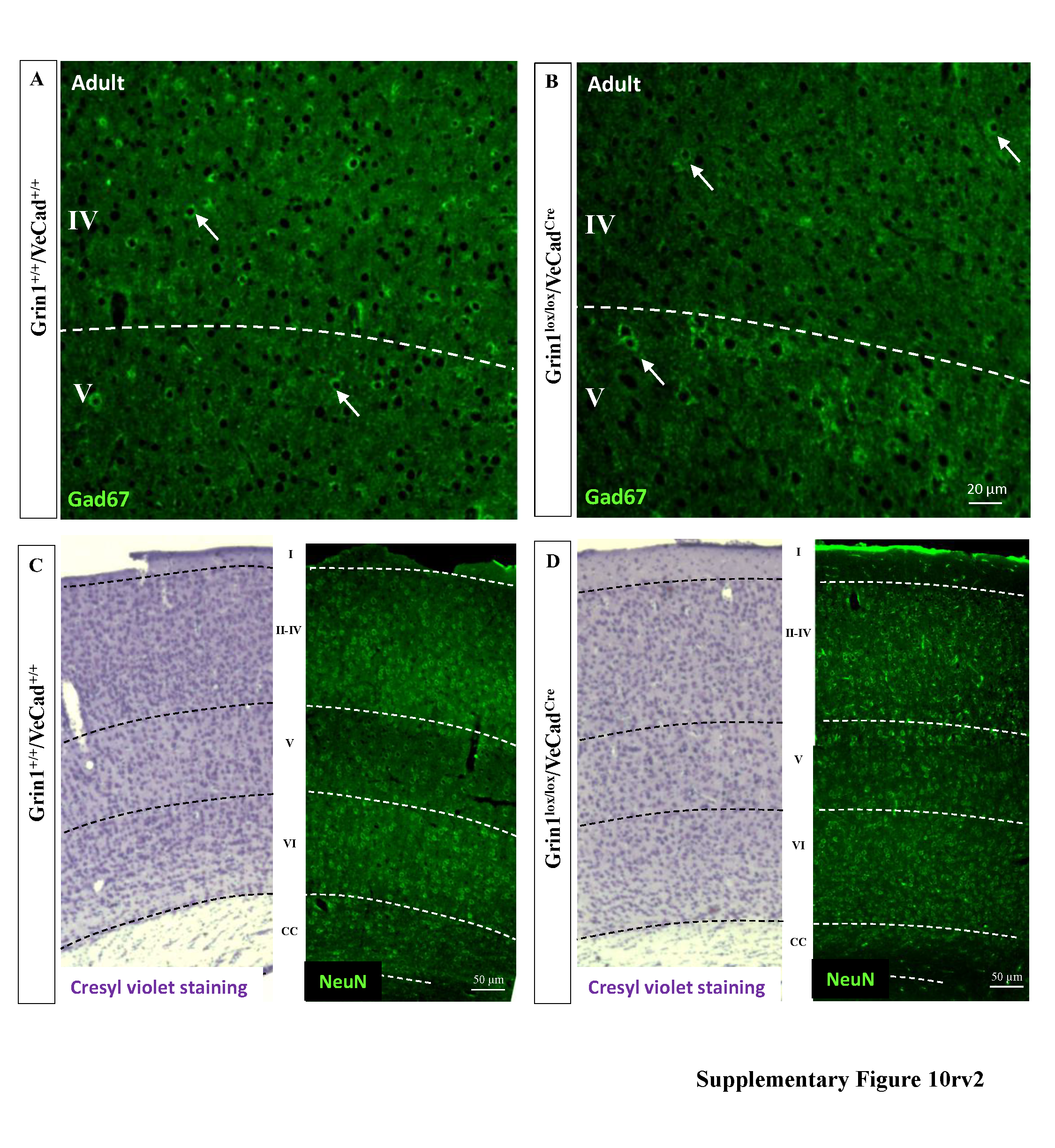

Supplement: Supplementary file 10 — Supplementary Fig. 10 Visualization at high magnification of Gad67-positive neurons in the layers IV and V of the neocortex from Grin1+/+/VeCad+/+ (A) and Grin1lox/lox/VeCadCre (B) mice. Note the depopulation of Gad67 immunoreactive cells in mutant mice. C,D Cresyl violet staining and NeuN immunoreactive cells visualizing the cortical layering in Grin1+/+/VeCad+/+ (C) and Grin1lox/lox/VeCadCre (D) in adult mice. Note the increased thickness of layer I and the decrease of Cresyl violet-stained cell bodies in Grin1lox/lox/VeCadCre mice. No layering differences were observed between inter- and intra-littermate control mice (data not shown) (TIFF 25455 kb) [file 18_2019_3248_MOESM10_ESM.tif]
