# Supplementary material for: Glutamate controls vessel-associated migration of GABA interneurons from the pial migratory route via NMDA receptors and endothelial protease activation
Source: Cell Mol Life Sci. 2019 Aug 7;77(10):1959–86. doi: 10.1007/s00018-019-03248-5 (PMC7229000; doi:10.1007/s00018-019-03248-5)
Supplement: Supplementary file 11 — Supplementary material 11 (DOCX 18 kb) [file 18_2019_3248_MOESM11_ESM.docx]

**Supplementary Table 1** Origin and characteristics of the primary antibodies used for the immunohistochemical and Western blot studies.

| **Antibodies** | **Trade reference** | **Purified species** | **Supplier** | **Dilution** | **Solution of incubation** |
| --- | --- | --- | --- | --- | --- |
| **ACTA2-FITC** | **IHC: F3777** | **Mouse** | **Sigma** | **1/200** | **1% BSA, 3% Triton X-100 in PBS** |
| CD31/PECAM | IHC: cat 550274 | Rat | BD-Pharmigen | 1/400 | 1% BSA, 3% Triton X-100 in PBS |
| Calretinin CR | IHC: cat 180211 | Rabbit | ThermoFisher Scientific | 1/400 | 1% BSA, 3% Triton X-100 in PBS |
| CRE recombinase | IHC: Ab190177 | Rabbit | Abcam | 1/400 | 1% BSA, 3% Triton X-100 in PBS |
| GFAP | IHC: Ab7260 | Rabbit | Sigma | 1/400 | 1% BSA, 3% Triton X-100 in PBS |
| **GFAP-Cy3**  **(for triple immunolabeling with DCX and CD31)** | **IHC: C9205** | **Mouse** | **Sigma** | **1/400** | **1% BSA, 3% Triton X-100 in PBS** |
| Doublecortin  DCX | IHC: Ab18723 | Rabbit | Abcam | 1/400 | 1% BSA, 3% Triton X-100 in PBS |
| DCX  (for GABA co-labeling) | IHC: Ab135349 | Mouse | Abcam | 1/400 | 1% BSA, 3% Triton X-100 in PBS |
| GABA | IHC: A2052 | Rabbit | Sigma-Aldrich | 1/400 | 1% BSA, 3% Triton X-100 in PBS |
| GFP | IHC: Ab6673 | Goat | Abcam | 1/400 | 1% BSA, 3% Triton X-100 in PBS |
| GluN1 | IHC: SC1467 | Goat | Santa Cruz | 1/400 | 1% BSA, 3% Triton X-100 in PBS |
| MMP-9 | IHC: PAB12714 | Rabbit | Abnova | 1/400 | 1% BSA, 3% Triton X-100 in PBS |
| Collagen IV  ColIV | IHC: Ab86042 | Mouse | Abcam | 1/400 | 1% BSA, 3% Triton X-100 in PBS |
| Somatostatin  SST | IHC: SC13099  WB: SC13099 | Rabbit | Santa Cruz | 1/400  1/1000 | IHC: 1% BSA, 3% Triton X-100 in PBS  WB: BSA (5% in TBST) |
| t-PA | IHC | Rabbit | Generous gift from R. Lijnen | 1/400 | 1% BSA, 3% Triton X-100 in PBS |
| β-Actin | WB: A5441 | Mouse | Sigma- Aldrich | 1/5000 | Milk  (5% in TBST) |
