# Supplementary material for: Glutamate controls vessel-associated migration of GABA interneurons from the pial migratory route via NMDA receptors and endothelial protease activation
Source: Cell Mol Life Sci. 2019 Aug 7;77(10):1959–86. doi: 10.1007/s00018-019-03248-5 (PMC7229000; doi:10.1007/s00018-019-03248-5)

**Supplementary Table 2** Sequences of the primers used for qRT-PCR experiments targeting the GluN1 (*Grin1*), GluN2A (*Grin2a*) and GluN2B (*Grin2b*) subunits of the NMDA receptor. *f*, forward; *r*, reverse. GAPDH was used as internal control.


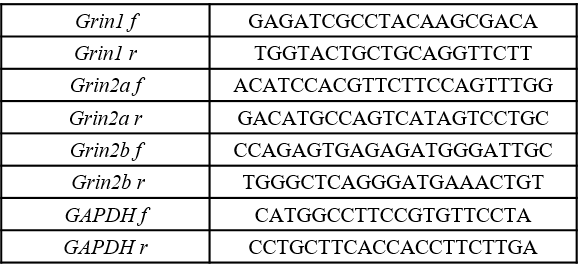

Supplement: Supplementary file 12 — Supplementary material 12 (DOCX 28 kb) [file 18_2019_3248_MOESM12_ESM.docx]
